# Cancer cell membrane-coated nanoparticles for bimodal imaging-guided photothermal therapy and docetaxel-enhanced immunotherapy against cancer

**DOI:** 10.1186/s12951-021-01202-x

**Published:** 2021-12-24

**Authors:** Qiaoqi Chen, Liang Zhang, Lin Li, Mixiao Tan, Weiwei Liu, Shuling Liu, Zhuoyan Xie, Wei Zhang, Zhigang Wang, Yang Cao, Tingting Shang, Haitao Ran

**Affiliations:** 1 Chongqing Key Laboratory of Ultrasound Molecular Imaging, Institute of Ultrasound Imaging, The Second Affiliated Hospital, Chongqing Medical University, No.76 Linjiang Road, Yuzhong District, Chongqing, 400010 People’s Republic of China; 2 Department of Ultrasound, The First Affiliated Hospital, Chongqing Medical University, No.1 Youyi Road, Yuzhong District, Chongqing, 400042 People’s Republic of China; 3 Department of Radiology, Chongqing University Cancer Hospital & Chongqing Cancer Institute & Chongqing Cancer Hospital, No. 181 Hanyu Road, Shapingba District, Chongqing, 400030 People’s Republic of China; 4 Chongqing General Hospital, University of Chinese Academy of Sciences, No.114 Longshan Road, Yubei District, Chongqing, 401121 People’s Republic of China

**Keywords:** Cocktail therapy, Photothermal therapy, Immunosuppressive tumor microenvironment, Homologous targeting, Nanomedicine

## Abstract

**Background:**

Mono-therapeutic modality has limitations in combating metastatic lesions with complications. Although emerging immunotherapy exhibits preliminary success, solid tumors are usually immunosuppressive, leading to ineffective antitumor immune responses and immunotherapeutic resistance. The rational combination of several therapeutic modalities may potentially become a new therapeutic strategy to effectively combat cancer.

**Results:**

Poly lactic-co-glycolic acid (PLGA, 50 mg) nanospheres were constructed with photothermal transduction agents (PTAs)-Prussian blue (PB, 2.98 mg) encapsulated in the core and chemotherapeutic docetaxel (DTX, 4.18 mg)/ immune adjuvant-imiquimod (R837, 1.57 mg) loaded in the shell. Tumor cell membranes were further coated outside PLGA nanospheres (designated “M@P-PDR”), which acted as “Nano-targeted cells” to actively accumulate in tumor sites, and were guided/monitored by photoacoustic (PA)/ magnetic resonance (MR) imaging. Upon laser irradiation, photothermal effects were triggered. Combined with DTX, PTT induced in situ tumor eradication. Assisted by the immune adjuvant R837, the maturation rate of DCs increased by 4.34-fold compared with that of the control. In addition, DTX polarized M2-phenotype tumor-associated macrophages (TAMs) to M1-phenotype, relieving the immunosuppressive TME. The proportion of M2-TAMs decreased from 68.57% to 32.80%, and the proportion of M1-TAMs increased from 37.02% to 70.81%. Integrating the above processes, the infiltration of cytotoxic T lymphocytes (CTLs) increased from 17.33% (control) to 35.5%. Primary tumors and metastasis were significantly inhibited when treated with “Nano-targeted cells”-based cocktail therapy.

**Conclusion:**

“Nano-targeted cells”-based therapeutic cocktail therapy is a promising approach to promote tumor regression and counter metastasis/recurrence.

**Graphical Abstract:**

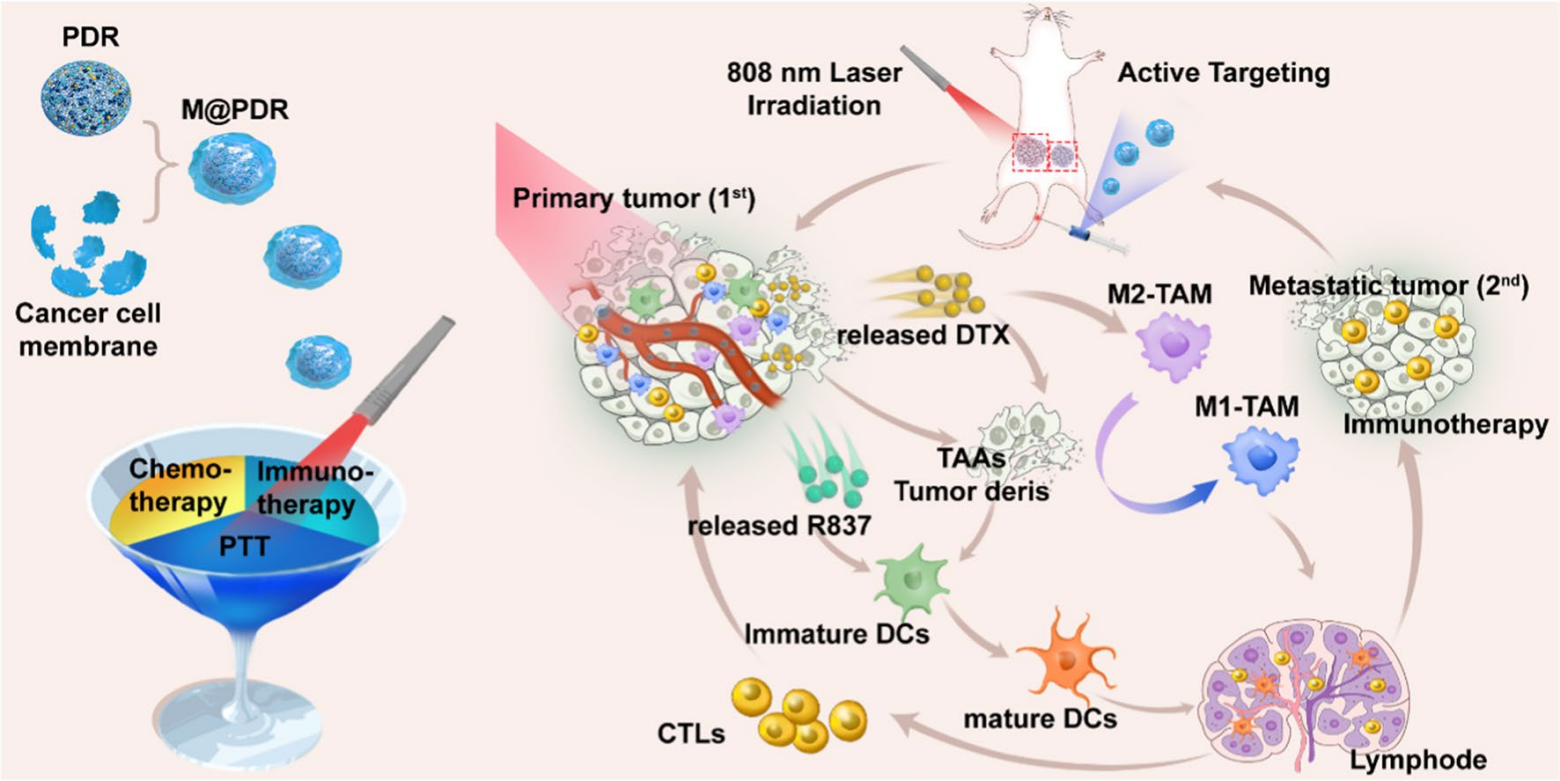

**Supplementary Information:**

The online version contains supplementary material available at 10.1186/s12951-021-01202-x.

## Introduction

Cancer is one of the leading causes of death, and extensive efforts have been made to suppress cancer progression [1]. Conventional oncologic methodologies, such as surgical resection, radiotherapy, and chemotherapy, may cause severe side-effects to normal tissues, and some patients may suffer from recurrence and metastases [2, 3]. Therefore, developing more effective therapeutic strategies is crucial. Because of the versatility and exclusive properties of nanomaterials, therapeutic techniques based on nanoparticles have been developed and have attracted increasing attention. However, relying on only one modality always has limitations in combating complicated metastatic lesions [4]. Seeking synergy among two or more approaches may provide insight into future improvements.

In the past decades, we have witnessed preliminary efficacy of emerging hyperthermia therapy (HTT) against malignant tumors [5, 6]. As one of the paradigms of HTT, photothermal therapy (PTT) takes advantage of localized photothermal transduction agents (PTAs), such as superparamagnetic nanoparticles which accumulate at the tumor site under the driving of magnetic field, to convert light energy into heat and subsequently raises the temperature of the tumor site, thereby inducing cancer cell death [7, 8]. Powered by nanotechnologies, PTT offers unparalleled advantages, such as noninvasiveness, controllable power of laser irradiation and extremely low toxicity to normal tissues [9–11]. As a classic PTA, Prussian blue (PB) nanoparticles are widely used in PTT due to their facile preparation process, low cost, good biocompatibility, and biosafety, as well as their good photothermal conversion efficiency. In addition, PB nanoparticles can also act as contrast agents for enhanced photoacoustic (PA) imaging and T1-weighted magnetic resonance (MR) imaging, which can provide guidance/monitoring for PTT [12, 13]. Although PTT can inhibit the growth of primary tumors to some extent, it has certain disadvantages, such as limited light penetration, which could result in inadequate tumor tissue ablation [14]. In fact, recurrences after hyperthermic ablation are common [15, 16], and urgently need to be addressed.

PTT has recently been discovered to not only effectively destruct the primary tumor, but also to activate an immune response that inhibits recurrence and metastasis [17, 18]. However, due to the infiltration of immunosuppressive cells, such as protumoral M2-phenotype tumor-associated macrophages (TAMs), solid tumors are usually immunosuppressive, which can lead to ineffective antitumor immune response and immunotherapeutic resistance. It is essential to improve the efficacy of immune response and alleviate the tumor microenvironmental immunosuppression [19, 20]. Dendritic cells (DCs) have been recognized as the most potent antigen-presenting cells (APCs), and the efficacy of T cells activation is mainly determined by the maturation stage of DCs. Therefore, immune adjuvants, which are non-specific immunopotentiators, are introduced to promote the maturation of DCs. Among various adjuvants, imiquimod (R837), a Toll-like receptor-7 agonist, has been demonstrated to significantly stimulate the maturation of DC cells and activate cytotoxic T cells (CTLs) [21, 22]. However, some also suggested that the immunosuppressive TME can cause dysfunction in CTLs, which further hinders the killing efficacy of CTLs. Recent studies have demonstrated that certain chemotherapeutic drugs, such as docetaxel (DTX), can attack tumor cells, prompt the release of TAAs, and effectively reverse the immunosuppressive TME by polarizing protumoral M2-phenotype TAMs to tumoricidal M1-phenotype TAMs [23, 24]. This is of great significance as M2 macrophages are widely acknowledged to be a major immunosuppressive population within tumors [25].

In the abovementioned modalities, although when applied individually, it may have certain drawbacks, for instance, PPT may lead to inadequate hyperthermia ablation and the immunosuppressive can hinder the immunotherapy. Fortunately, PTT combined with chemotherapy and immunotherapy could seemingly compensate for the drawbacks of each other, and achieve a synergistic therapeutic outcome [23, 26]. To rationally integrate these modalities, nanoplatforms with a multimodal therapeutic potential need to be developed, and off-target delivery must be minimized to ensure the efficient accumulation of these nanoplatforms into tumor tissues [9, 27]. Poly lactic-co-glycolic acid (PLGA) is a biodegradable and biocompatible polymer that has been widely used in biomedical research, it has also been approved by the U.S. Food and Drug Administration (FDA) [28, 29]. And PLGA can easily load both hydrophilic and hydrophobic drugs, which makes PLGA one of the best candidates for smart drug delivery systems (DDSs) [30]. It is worth mentioning that the ultimate goal of DDS is to successfully deliver the therapeutic drug to the tumor site, which calls for a high standard of colloidal stability and prolonged blood circulation. In tumors, multi-cancer-cellular aggregation are usually ascribed to surface adhesion molecules (*eg.*, epithelial cell adhesion molecule (EpCAM) and galectin-3) expressed on the surface of cancer cell membranes with homologous adhesion domains [31–33]. In other words, cancer cell membranes possess the ability to homologously bind to cancer cells. Therefore, cancer cell membrane-coated nanoparticles can be ideal drug carriers as they are expected to bind specifically with cancer cells [34]. In addition, due to the presence of antigen retention, cancer cell membrane-coating could significantly circumvent the in vivo immune clearance by reducing the uptake of the reticuloendothelial system (RES) [35–37].

**Scheme 1:**
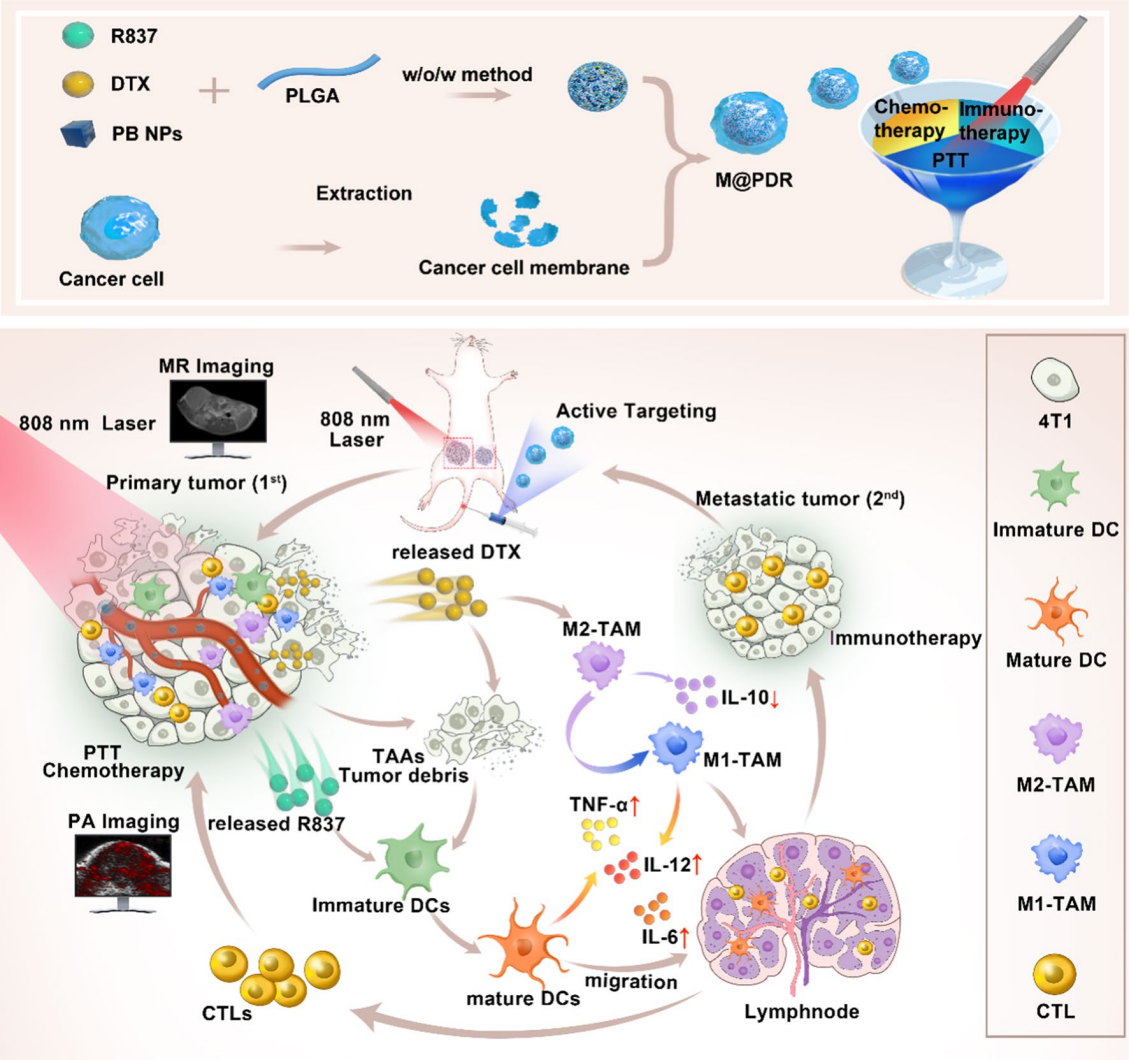
Schematic diagram of the homologous targeted tumor cocktail therapy based on M@P-PDR “Nano-targeted cells”

Inspired by these findings, we report a homologous targeted cocktail therapy that integrates several therapeutic modalities. Just as a cocktail is a rational combination of several different wines to obtain the best taste, this study integrates several therapeutic modalities to achieve the best therapeutic effect. In this study, PB nanoparticles were selected as PTAs and acted as contrast agents for enhanced PA and T1-weighted MR bimodal imaging, providing guidance/monitoring during treatment. Cell membrane-coated PLGA nanospheres are constructed with PB encapsulated in the core and DTX/R837 loaded in the shell (designated “M@P-PDR”). M@P-PDR nanospheres act as “Nano-targeted cells” to actively accumulate in tumor sites due to the homologous targeting capability. Upon laser irradiation, combined with DTX, PTT induces in-situ tumor eradication, releasing TAAs, and further enhancing tumor cell immunogenicity. In addition, DTX can relieve the immunosuppressive TME by polarizing protumoral M2-phenotype oncogenic TAMs to tumoricidal M1-phenotype oncogenic TAMs. Furthermore, immune adjuvant R837 promotes the maturation of DCs, which can more effectively present tumor-associated antigens (TAAs) and further stimulate the host immune response. Cocktail therapy, namely PTT combined with DTX-enhanced immunotherapy, creates a “doomsday storm” for tumors (Scheme 1). This study provides a feasible approach to promote tumor regression and counter metastasis/recurrence. Considering that all ingredients in these “Nano-targeted cells” are FDA-approved and that their biosafety/biocompatibility has been systematically studied and evaluated, these as-synthesized nanospheres hold great potential for further clinical translation.

## Materials and methods

### Materials

All reagents used in this work were of analytical grade. Citric acid, FeCl_3_, and K_4_[Fe(CN)_6_] were purchased from Shanghai Jingchun Biological Technology Co., Ltd. (Shanghai, China). PLGA (lactide: glycolide = 50:50, PLGA 12,000 Da Mw) was obtained from Shandong Daigang Biology Engineer Corp. (China). Imiquimod (R837), docetaxel (DTX), Poly (vinyl alcohol) (PVA 25,000 Mw), calcein-AM (CAM), propidium iodide (PI), 1,1′-dioctadecyl-3,3,3′,3′-tetramethylindotricarbocyanine iodide (DiR) and fluorescence dyes 1,1′-dioctadecyl-3,3,3′,3′ tetra-methylindocarbocyanine perchlorate (DiI) were purchased from Sigma-Aldrich (Shanghai, China). Membrane protein extraction kits, phenylmethanesulfonyl fluoride (PMSF), penicillin–streptomycin solution, and trypsin were purchased from Beyotime (Shanghai, China). Trichloromethane (CHCl_3_) was purchased from Chongqing Chuandong Chemical Corp. (Chongqing, China). Cell Counting Kit-8 (CCK-8) was obtained from MedChemExpress (Monmouth Junction, NJ, USA). Enzyme-linked immunosorbent assay (ELISA) kits including mouse IL-6, L-12, TNF-α and IL-10 were purchased from Meimian Industrial Co., Ltd. (Jiangsu, China).

### Synthesis of P-PDR

The synthesis of PB nanoparticles is described in detail in the supporting information. PLGA nanospheres loaded with PB nanoparticles, DTX and R837 (designated P-PDR) were synthesized using a typical double-emulsion method (water/oil/water). Briefly, DTX (6 mg), R837 (2 mg) and PLGA (50 mg) were dissolved in 2 mL of trichloromethane (CHCl_3_), followed by the addition of 400 μL of PB nanoparticles (20 mg mL^−1^). Then, the mixture was emulsified by using an ultrasonic probe (Sonics & materials, Inc., USA) at 78 W for 2 min to form the first emulsion. Subsequently, 6 mL of PVA solution (4% w/v) was added to the above emulsion for the second emulsion with sonication power at 65 W. Afterward, isopropanol solution (6 mL, 2% w/v) was dissolved in the second emulsion for magnetic stirring. Finally, after centrifugation at 10, 000 g for 6 min, the P-PDRs were collected from the slurry and stored at 4 °C for further use. PLGA nanospheres loaded with different ingredients, such as P-P (PLGA loaded with PBs), P-DR (PLGA loaded with DTX and R837), P-PD (PLGA loaded with PBs and DTX), and P-PR (PLGA loaded with PBs and R837) were prepared by the same process as described above by replacing the cargo. DiI or DiR-labeled PLGA nanospheres were fabricated in the same manner, except that DiI or DiR was added to the CHCl_3_ mixture.

### Extraction of cancer cell membrane and synthesis of M@P-PDR

Murine breast cancer cells (4T1) were purchased from Zhongqiaoxinzhou Biotechnology Co., Ltd. (Shanghai), and cultured in Roswell Park Memorial Institute (RPMI) 1640 complete medium (Zhongqiaoxinzhou Biotechnology Co., Ltd., ZQ-Z201) containing 10% fetal bovine serum and 1% penicillin/streptomycin in a humidified incubator at 37 °C with 5% CO_2_. The cell membrane was extracted using a membrane protein extraction kit from Beyotime Biotechnology. Cancer cell membrane-coated PLGA nanospheres were constructed according to a previous method with slight modifications [38–40]. Briefly, a 4T1 cell membrane suspension (1 mg mL^−1^) was mixed with P-PDR nanospheres under probe sonication (2 min, 40 W). Afterward, the mixture was centrifuged (10,000 g, 4 ℃, 10 min) and the slurry was washed twice to obtain M@P-PDR nanospheres.

### Characterization of M@P-PDR

The morphology of M@P-PDR was observed with a transmission electron microscopy (TEM, Hitachi H-7600, Japan) and a scanning electron microscopy (SEM, Zeiss SUPRA™ 55, Germany). The protein content of M@P-PDR was analyzed by the SDS-PAGE method. The particle size distribution and zeta potential of M@P-PDR were measured with a dynamic laser scattering (DLS) particle sizer (ZEN3600, Malvern Instruments, UK). PB nanoparticles, R837, DTX, PLGA, P-P, P-PDR and M@P-PDR were analyzed using Fourier transform infrared spectroscopy (FTIR, Nicolet iS10, Thermo Scientific Co. Ltd., MA, USA). The contents of R837 and DTX in M@P-PDR were measured by liquid chromatography-mass spectrometry (LC–MS, chromatograph: UltiMate 3000 RS, mass spectrometer: TSQ Quantum GC, China). The UV absorption spectrum of PB was obtained by a spectrophotometer (UV-3600, Shimadzu, Japan). The loading efficacy of PB, R837 and DTX was calculated by the following formula:

Loading efficacy (%w/w) = (mass of PB/R837/DTX in M@P-PDR) / total mass of PB, R837 or DTX input × 100%

### In vitro photothermal performance and PA/MR bimodal imaging of M@P-PDR

To study the photothermal performance of M@P-PDR, the temperature changes of M@P-PDR (with concentration at 1, 2, 3, 4 and 5 mg mL^−1^) after 808 nm laser irradiation were monitored by an infrared thermal camera (Fotri226, Shanghai, China). The laser power intensity was set at 1.5 W cm^−2^, 5 min. Besides, 5 mg mL^−1^ of M@P-PDR nanospheres were irradiated with different power intensities (0.75, 1.00, 1.25 and 1.50 W cm^−2^) for 5 min. In addition, the photothermal stability of M@P-PDR was analyzed by exposing M@P-PDR to five cycles of laser irradiation on/off. For the in vitro PA imaging, M@P-PDR nanospheres at a concentration of 2 mg mL^−1^ was scanned by PA laser with the excitation wavelength ranging from 680 to 970 nm (interval = 5 nm) to detect the optimum excitation wavelength using a PA imaging system (Vevo LAZR, CA). Then, M@P-PDR nanospheres at different concentrations (2, 4, 6, 8 and 10 mg mL^−1^) were imaged with the optimal settings (λ = 740 nm). The acquired PA images were then analyzed by Vevo LAZR software to quantify the PA signal intensities within the region of interest (ROI). For T1-weighted MR imaging, different concentrations (1.25, 2.5, 5, 10 and 20 mg mL^−1^) of M@P-PDR nanosphere were placed in 2 mL Eppendorf tubes for MR imaging using an MR imaging scanning device (Philips Achieva 3.0 T, Netherland). T1-weighted images of all samples were obtained using the following parameters: fast field echo (FFE), repetition time (TR) = 494 ms, echo time (TE) = 10 ms, flip angle = 90°, field of view (FOV) = 190 mm, and slice thickness = 1.0 mm. the T1 signal intensities within the ROI were also measured.

### Validation of cell adhesion molecules, homologous targeting capability and cytotoxicity evaluation of M@P-PDR

The expression of cell adhesion molecules including EpCAM, and galectin-3 in cancer cell, cancer cell membrane, M@P-PDR nanospheres and P-PDR nanospheres samples was also analyzed by Western blot. To investigate the specificity of M@P-PDR nanospheres to target homologous 4T1 breast cells, the targeting ability of M@P-PDR nanospheres to 4T1 cells, MDA-MB-231 human breast cancer cells and SKBR3 human breast cancer cells has also been evaluated. 4T1 cells, MDA-MB-231 cells and SKBR3 cells were seeded in confocal-specific dishes and cultured for 24 h, and then DiI-labeled M@P-PDR nanospheres suspensions were added to the above dishes at an equivalent PLGA concentration of 50 μg mL^−1^. After 2 h of incubation, the nuclei were stained with DAPI for confocal laser scanning microscope (CLSM, LSM710. Carl Zeiss, Germany) observation. 4T1 cells were further treated with DiI-labeled M@P-PDR or P-PDR nanospheres suspensions (equivalent PLGA concentration: 50 μg mL^−1^), the nuclei were stained with DAPI for CLSM observation after various incubation times (0.5, 1, 2, 3, and 4 h). Flow cytometry (BD FACSVantage SE, USA) was also carried out for quantitative analysis.

To observe the cytotoxicity of M@P-PDR against 4T1 breast cancer, 2 × 10^4^ cells per well were cultured in a 96-well for 24 h. Then, different concentrations of P-PDR and M@P-PDR (equivalent PLGA concentrations at 20, 40, 60, 80, 100, 200 and 400 µg mL^−1^) dispersed in RPMI 1640 medium were added and cocultured for 24 h. And then the cell viabilities were tested via a typical CCK-8 assay. Additionally, 4T1 cells seeded in 96-well plates (2 × 10^4^ cells per well) were randomly divided into seven groups including (i) control group, (ii) laser-only group, (iii) M@P-PDR group, (iv) M@P-DR + laser group, (v) M@P-PR + laser group, (vi) P-PDR + laser group, and (vii) M@P-PDR + laser group. The cells in various groups were treated with the corresponding nanospheres (the equivalent PLGA concentration is 400 μg mL^−1^) for another 4 h, followed by 808 nm laser irradiation (1.5 W cm^−2^, 5 min). Then the cell viabilities were tested by a typical CCK-8 assay. For flow cytometry assessments, 4T1 cells were cultured in 6-well plates (8 × 10^4^ cells per well) overnight. After various treatments, the proportions of apoptosis in each group were analyzed. Furthermore, cell viabilities were also visualized by CAM /PI staining.

### In vitro DC maturation analysis

BALB/c mice bone marrow-derived DCs were purchased from Otwo. Biotech. Inc. (Shenzhen, China). To evaluate the in vitro DCs activation, a transwell system was used. Initially, 4T1 cells were seeded in the upper chambers, and the immature DCs were seeded in the lower plates. They were randomly divided into six groups including (i) control group, (ii) M@P-PDR group, (iii) M@P-PD + laser group, (iv) M@P-PR + laser group, (v) P-PDR + laser group, and (vi) M@P-PDR + laser group, and received the corresponding treatment, respectively. Then the 4T1 cells in the upper chambers were harvested and incubated with the lower plates for another 24 h. Finally, DCs were collected and stained with anti-CD11c-FITC, anti-CD86-PE and anti-CD80-APC (eBioscience, Thermo Science, USA) for flow cytometry analysis. Otherwise, the supernatant was assayed by the ELISA kit for the detection of IL-6, IL-12 and TNF-α.

### Animal models

Female BALB/c mice (6 weeks old) were purchased from Enswell Biotechnology Ltd (Chongqing, China). All experimental protocols in this study were performed in the Chongqing Medical University Laboratory Animal Center and all protocols were approved by the Animal Ethics Committee of the Second Affiliated Hospital of Chongqing Medical University (2018–43). To inoculate the 4T1 breast cancer model, 4T1 cells (1.2 × 10^6^ cells per mouse) suspended in RPMI-1640 medium were subcutaneously injected into the fifth mammary fat pad on the left side.

### In vivo biosafety and biodistribution of M@P-PDR

To evaluate the in vivo biosafety of these M@P-PDR “Nano-targeted cells”, healthy BALB/c mice were intravenously administrated with M@P-PDR nanospheres suspension (3 mg mL^−1^, 200 μL per mouse). Mice were sacrificed at 1 d, 3 d, 7 d, 15 d and 30 d (n = 5) post the injection, and then blood samples were collected for hematology analysis and serum biochemical tests, respectively. Major organs (heart, liver, spleen, lung, and kidney) were subjected to H&E staining. Mice injected with saline were set as control.

To explore the biodistribution and in vivo targeting behavior of these “Nano-targeted cells”, tumor-bearing mice were randomly divided into two groups (n = 3), they were then injected with DiR-labeled M@P-PDR or P-PDR nanospheres (equivalent PLGA concentration at 3 mg mL^−1^, 200 µL), respectively. Then, these mice were subjected to an in vivo fluorescence imaging system at various time intervals post above administration to record the DIR fluorescence imaging. In the meantime, the corresponding fluorescence intensities were analyzed. Finally, animals were sacrificed to harvest the major organs (heart, liver, spleen, lung, and kidney) and tumors for ex vivo fluorescence evaluation.

### In vivo MR/PA bimodal imaging

4T1 tumor-bearing mice were randomly divided into two groups (n = 3), each mouse was injected with M@P-PDR or P-PDR nanospheres (equivalent PLGA concentration at 3 mg mL^−1^, 200 µL), respectively. The PA images in the tumor region were acquired with a prolonged post-injection period (0, 1, 2, 4, 6, 8, and 24 h). Likewise, the corresponding PA intensities were quantitatively analyzed. T1-weighted MR imaging was also carried out after intravenous injection of these developed M@P-PDR. The greyscale images were converted to pseudo-color using MATLAB (2016). The signal intensities (SI) of the tumor tissues were measured, and the percentage of signal intensity enhancement (PSIE) was simultaneously calculated. PSIE was calculated as follows: (SIpost—SIpre)/ SIpre × 100%.

### In vivo photothermal performance and tumor growth inhibition evaluation

To mimic distant tumors, after 6 days of primary tumor incubation (Day 7), equivalent 4T1 cells were subcutaneously injected into the right mammary fat pads at day-1. Then, all tumor bearing-mice were randomly divided into eight groups (n = 5) including: (i) saline group (control), (ii) M@P-PDR group, (iii)M@P-DR + laser group, (iv) M@P-P + laser group, (v) M@P-PR + laser group, (vi) M@P-PD + laser group, (vii) P-PDR + laser group, and (viii) M@P-PDR + laser group. The mice were intravenously injected with the corresponding nanospheres (equivalent PLGA concentration at 3 mg mL^−1^, 200 µL). Eight hours after the injection, the tumors were irradiated by an 808 nm laser (1.5 W cm^−2^, 10 min). The temperature changes in tumor areas were recorded by a thermal camera. On the 3^rd^ day posttreatments, one mouse in each group was sacrificed for primary tumor and major organs (heart, liver, spleen, lung, and kidney) dissection. Then, H&E staining and examination were performed. Besides, the tumor tissues were further stained with terminal deoxynucleotidyl transferase dUTP nick end labeling (TUNEL) and heat shock 70 kDa protein (HSP70). On Day 9, distant tumors were collected for proliferating cell nuclear antigen (PCNA) staining. To monitor tumor progression, the mice were photographed, and the tumor volume changes were measured. During the treatment periods, bodyweight of the mice was also recorded.

### Analysis of infiltrating immune cells

To detect the in vivo immune response, 4T1 xenotransplant tumors were divided into eight groups and received treatments identical to in vivo therapeutic evaluation. On the 9th day, both primary tumors and distant metastases were harvested for single-cell suspensions fabrication. The prepared cells were further stained with CD11c-FITC (eBioscience, Catalog: N418), CD86-PE (eBioscience, Catalog: GL1), CD80-APC (eBioscience, Catalog: 16-10A1), F4/80-APC (Biolegend, Catalog: BM8), CD11b-PE (Biolegend, Catalog: M1/70), CD80-FITC (Biolegend, Catalog: 16-10A1), CD206-FITC (Biolegend, Catalog: C068C2), CD3-FITC (Biolegend, Catalog:17A2), CD8a-APC (Biolegend, Catalog: QA17A07) and CD4-PC5.5 (Biolegend, Catalog: RM4-5) antibody and then analyzed by flow cytometry. Serum was collected from different groups of mice, and cytokines including IL-6, IL-12, TNF-α and IL-10 were analyzed using ELISA kits according to the manufacturer's protocols. In addition, immunofluorescence staining was further conducted to investigate the infiltrating immune cells in tumor tissues.

### Statistical analysis

Data are expressed as mean ± standard deviation, and the significance of the differences between the two groups was analyzed using the Student's two-tailed t-test (*p < 0.05, **p < 0.01, ***p < 0.001, ****p < 0.0001).

## Results and discussion

### Design, synthesis and characterization of M@P-PDR

**Fig. 1:**
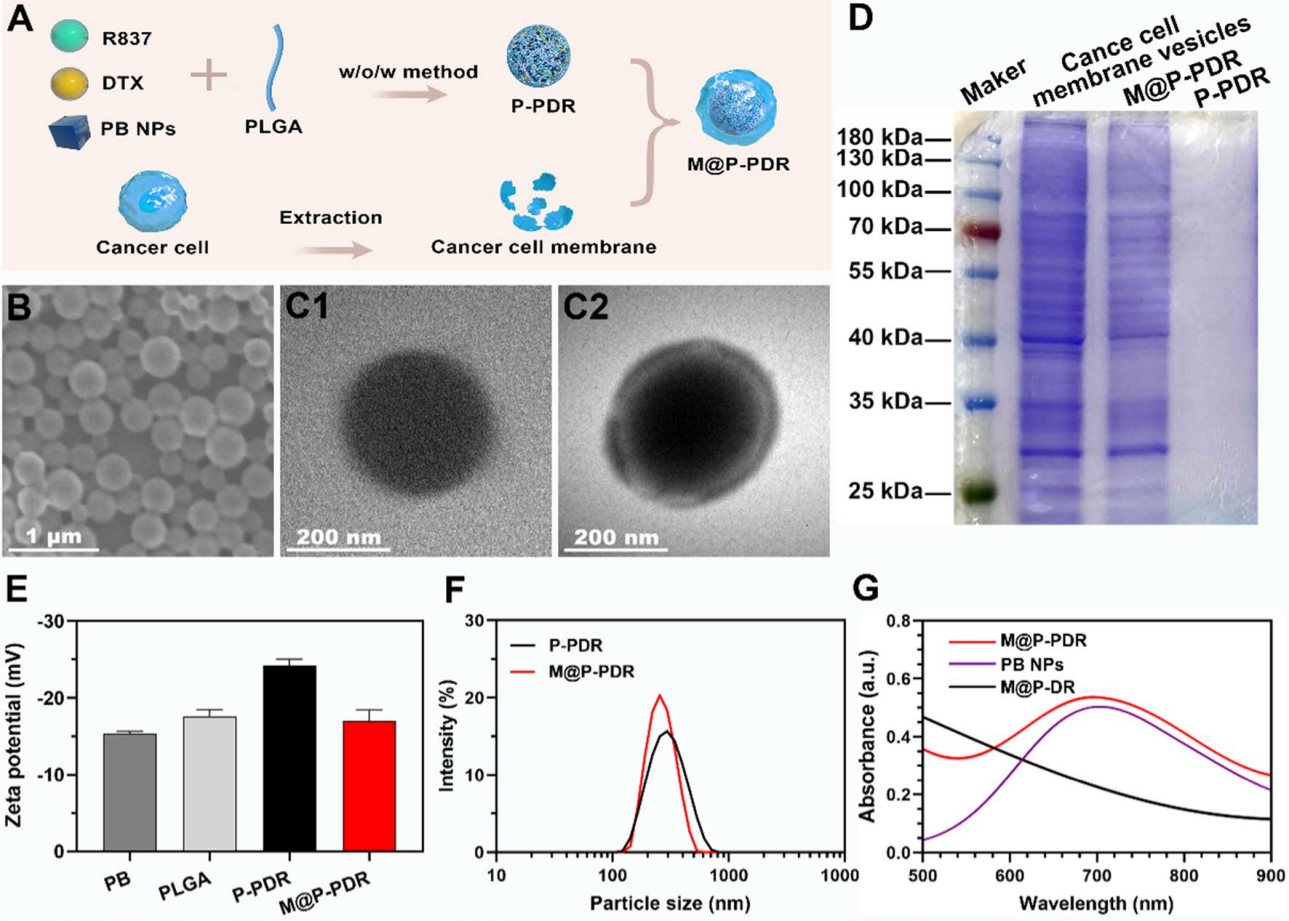
Characterization of the M@P-PDR nanospheres. **A** Schematic illustration of the synthetic process for M@P-PDR nanospheres. **B** SEM image of M@P-PDR. **C1** TEM image of P-PDR; **C2** TEM image of M@P-PDR. **D** SDS-PAGE protein analysis results of cancer cell membrane vesicles, M@P-PDR and P-PDR. **E** Zeta-potential of PB, PLGA, P-PDR and M@P-PDR nanospheres (n = 3). **F** DLS results of P-PDR and M@P-PDR nanospheres. **G** UV–Vis-NIR spectra of PB NPs, M@P-DR and M@P-PDR suspensions

M@P-PDR nanospheres, unique “Nano-targeted cells”, were constructed by coating P-PDR nanospheres with cancer cell membranes with homologous targeting capability (Fig. 1A). Initially, P-PDR nanospheres were prepared by a typical double- emulsion method (water/oil/water), which encapsulates hydrophobic drugs in the PLGA shell layer and hydrophilic drugs in the core of PLGA nanospheres [41–43]. PB nanoparticles are hydrophilic drugs and can be dispersed well in aqueous solution (Additional file 1: Fig. S1). Both R837 and DTX are lipid-soluble drugs. Therefore, PB is encapsulated in the core of PLGA nanospheres, while R837 and DTX are present in the shell. Coextrusion was the most adopted method to coat nanoparticles with membrane. Due to the fluidity of the cell membrane, the mechanical force exerted by the extrusion process promote the nanoparticles to be encapsulated by the phospholipid layer [33, 44]. However, this approach is a tedious and time-consuming process. Fortunately, sonication is an effective alternative to extrusion and used to prepare M@P-PDR nanospheres in this study [38]. Cell membranes would be destroyed by ultrasonic waves and the fragments could be self-assembled around the nanospheres. This one-step fabrication is facile approach. It has been found that repulsion between negatively charged nanoparticle-core and cell membrane-shell allows successful membrane coating with a “right-side-out” membrane topological manner orientation [45, 46]. The SEM image indicated that M@P-PDR displayed a uniform and spherical morphology (Fig. 1B). M@P-PDR “Nano-targeted cells” showed a more obvious coating than P-PDR (Fig. 1C), which could be ascribed to coverage by cancer cell membranes. Sodium dodecyl sulfate–polyacrylamide gel electrophoresis (SDS–PAGE) was used to further analyze the protein composition of these M@P-PDR “Nano-targeted cells”, and the results showed that M@P-PDR nanospheres had almost the same protein composition as the original 4T1 cell membrane (Fig. 1D), which further demonstrated the success of cell membrane coating. Excitingly, the P-PDR nanospheres are negatively charged (Fig. 1E), which ensures the orientation of the cell membrane during the coating process, thereby maintaining the biological function of the cell membrane [46]. The zeta potentials of M@P-PDR nanospheres was − 17.0 ± 1.40 mV, which could potentially prolong blood circulation and benefit other applications in the biological milieu [47].

Dynamic light scattering (DLS) showed that the average hydrodynamic diameter of the nanospheres slightly increased from 297 nm to 326.4 nm after cell membrane coating (Fig. 1F). We next analyzed the PB nanoparticles, drugs (R837 and DTX), PLGA, P-P, P-PDR and M@P-PDR by FTIR. As shown in Additional file 1: Fig. S2, PB, P-P, P-PDR and M@P-PDR have characteristic absorption peaks at 2090 cm^−1^, which is unique to PB nanoparticles, demonstrating that PB has been successfully encapsulated in PLGA [43]. M@P-PDR only showed absorption peaks which also appeared in PB nanoparticles, PLGA, DTX, R837 and P-PDR, but no other new characteristic absorption peaks were observed, further indicating that the binding between the components of the nanoparticles is physical rather than chemical. Compared to the UV–Vis spectrum of M@P-DR, the spectrum of the M@P-PDR suspension presented a characteristic absorption band of PB at 700 nm (Fig. 1G), indicating the successful loading of PB nanoparticles in M@P-PDR. The loading efficacies of PB nanoparticles, R837 and DTX were calculated to be 37.28%, 78.57% and 69.67%, respectively, according to the standard curves (Additional file 1: Fig. S3A, B) and liquid–mass spectrometry analysis (Additional file 1: Fig. S3C, D). Such high loading capacities for these drugs demonstrated that PLGA nanospheres have great potential as promising nanocarriers for drug delivery, which has also been reported by many previous studies [48, 49].

### In vitro photothermal performance and PA/MR bimodal imaging of M@P-PDR

**Fig. 2:**
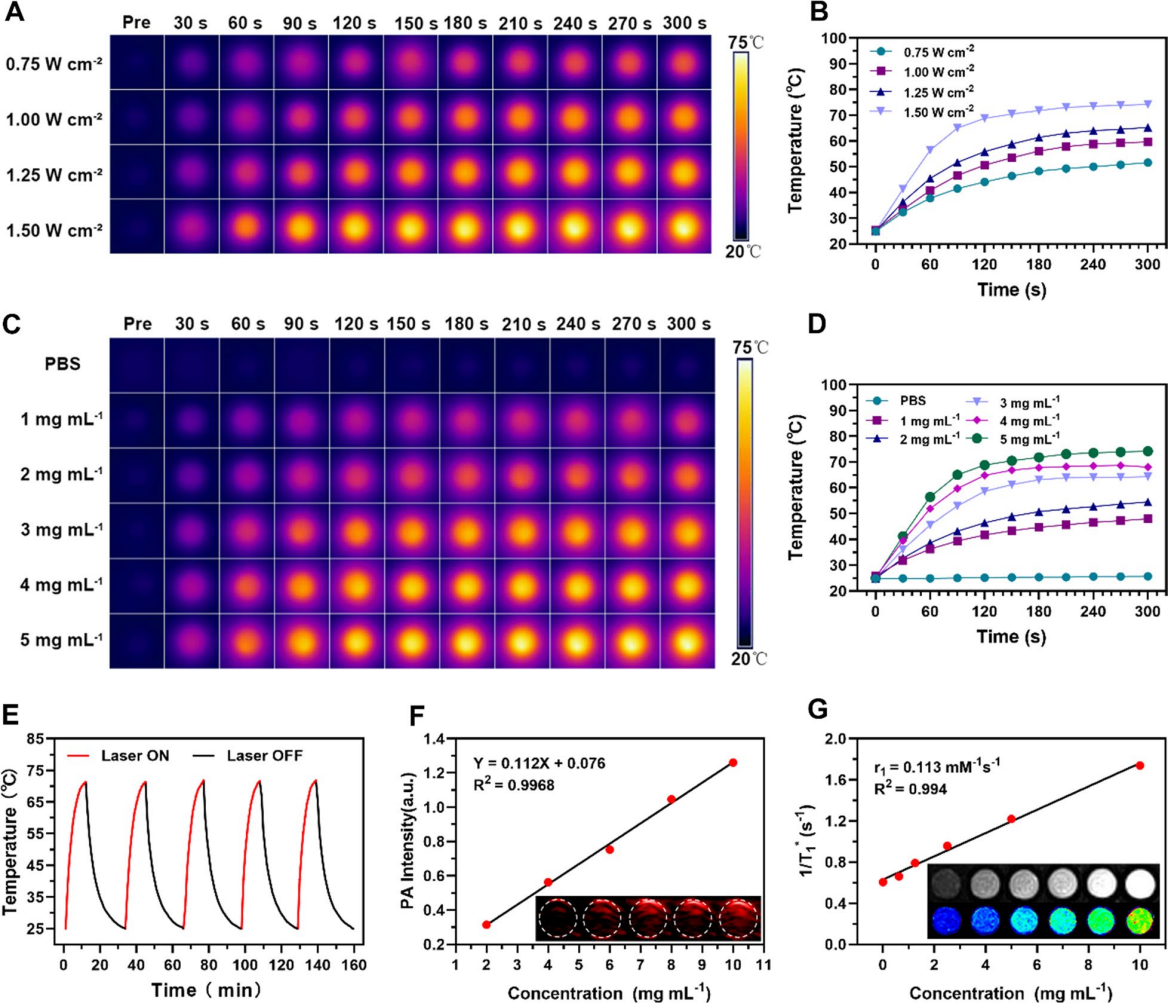
**A** Infrared thermal images of M@P-PDR at a concentration of 5 mg mL^−1^ under 808 nm laser irradiation at different power densities (0.75, 1.00, 1.25 and 1.50 W cm^−2^), and **B** the corresponding temperature–time curves of M@P-PDR at different power densities. **C** Infrared thermal images of M@P-PDR at different concentrations (0, 1, 2, 3, 4 and 5 mg mL^−1^) under 808 nm laser (1.5 W cm^˗2^, 5 min) irradiation, and **D** the corresponding photothermal temperature–time curves of M@P-PDRs at different concentrations. **E** Temperature change curves of M@P-PDR over five laser irradiation on/off cycles. **F** Linear relationship between PA intensities and M@P-PDR concentrations, and the corresponding in vitro PA images (inset). **G** T1 relaxation rate of M@P-PDR and the corresponding in vitro MR images (inset)

The distinctive absorbance of PB nanoparticles in the NIR region indicated the potential for M@P-PDR to boost photothermal therapeutics [50]. Therefore, the in vitro photothermal performance of M@P-PDR was systemically studied. The photothermal conversion of M@P-PDR nanospheres was evaluated at different laser power densities (0.75, 1.00, 1.25 and 1.50 W cm^˗2^) and different M@P-PDR concentrations (1, 2, 3, 4 and 5 mg mL^˗1^). Significant laser-power-dependent (Fig. 2A, B) and concentration-dependent (Fig. 2C, D) photothermal effects were observed. Moreover, excellent photothermal heating/cooling-cycling stability was also demonstrated (Fig. 2E). On this ground, M@P-PDRs can be used as PTCAs for subsequent PTT.

The high sensitivity and high spatial resolution of PA imaging facilitate the visualization of nanocarriers in vivo [51]. The multiwavelength PA signal spectrum of M@P-PDR nanospheres showed that 740 nm was the optimal wavelength for PA imaging (Additional file 1: Fig. S4). As shown in Fig. 2F, the PA signal intensities of M@P-PDR suspensions increased in a significant concentration-dependent manner. MR imaging performance was also investigated. As shown in Fig. 2G (inset), the brightness of the T1-weighted MR images increased with the concentration of M@P-PDR nanospheres, and the pseudo-colored T1-mapping images also showed the same tendency. The relaxation rate (R1 value) was calculated to be 0.113 mM^−1^ s^−1^ by measuring the relaxation time (Fig. 2G). With the enhanced PA/MR dual-modal imaging capacity, the metabolic profiles of these M@P-PDR nanospheres at tumor sites can be visualized, providing guidance/monitoring for subsequent cocktail therapy.

### Biocompatibility assay of M@P-PDR

As a prerequisite for any clinical development, the biocompatibility of M@P-PDRs was investigated both in vitro and in vivo. First, the cytotoxicity of M@P-PDR and P-PDR nanospheres toward 4T1 cells was evaluated using a standard CCK-8 assay. After 24 h of coincubation, both M@P-PDR and P-PDR nanospheres showed negligible toxicity to 4T1 cells when the PLGA concentration was lower than 400 μg mL^−1^ (Additional file 1: Fig. S5). To further investigate the biocompatibility of M@P-PDR, the in vivo acute and relatively long-term toxicity of M@P-PDR was evaluated in healthy BALB/c mice. Routine blood tests and serum biochemical assays were performed on Day 1, 3, 7, 15 and 30 after the intravenous administration of M@P-PDR (Additional file 1: Fig. S6A, B). Compared with the reference range of hematology data, all indicators of the treated mice and the control group remained at normal levels. In addition, the major organs (heart, liver, spleen, lung and kidney) were collected for H&E staining (Additional file 1: Fig. S6C), and negligible histomorphological or pathological changes were observed. All these results strongly demonstrated the ideal high biocompatibility of M@P-PDR nanospheres as a multitasking therapeutic agent, providing great potential for their further clinical translation.

### In vitro homologous targeting capacity of M@P-PDR

**Fig. 3:**
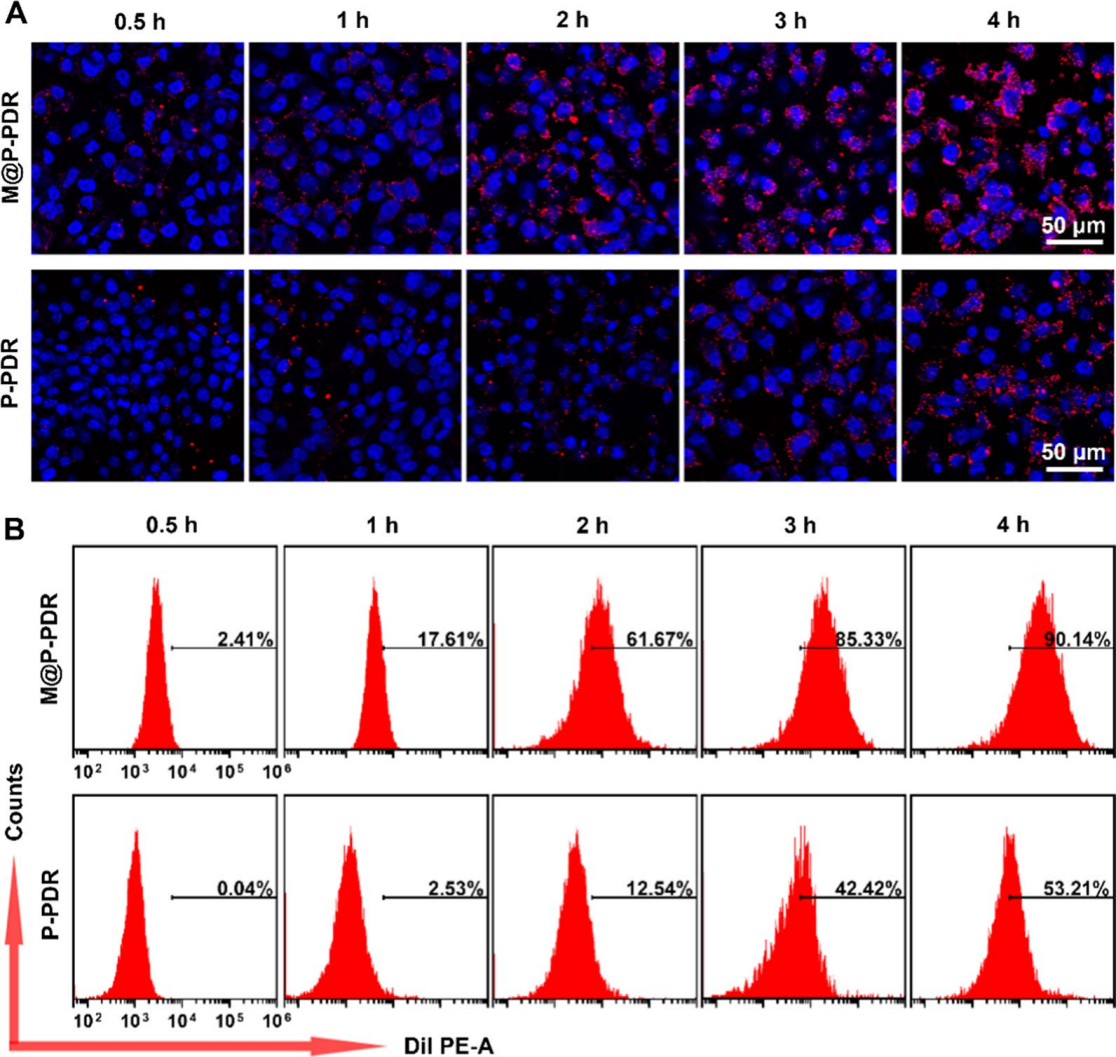
**A** CLSM images of 4T1 cells treated with M@P-PDR and P-PDR nanospheres for different times (0.5, 1, 2, 3 and 4 h), respectively (the blue indicates nucleus stained with DAPI, the red indicates M@P-PDR or P-PDR nanospheres stained with DiI), and **B** the corresponding flow cytometry quantitative analyses

The effective intracellular uptake of M@P-PDR nanospheres is the key to their therapeutic efficacy. Functionalized by adhesion proteins of cancer cells on the surface, cancer cell biomimetic nanoplatforms are expected to exhibit specific homologous targeting capacity [42, 52, 53]. Western blotting results showed the presence of these homologous binding adhesion molecules (EpCAM and galectin-3) on M@P-PDR (Additional file 1: Fig. S7), which could achieve specific recognition and binding between source cells specifically targeting M@P-PDR and cancer cells through a homologous binding mechanism. To investigate the specificity of M@P-PDRs to homologous 4T1 cells, the targeting capability of M@P-PDRs to 4T1 cells, MDA-MB-231 cells and SKBR3 cells was verified by CLSM and flow cytometry. The results showed that the 4T1 group had the highest uptake efficiency and fluorescence intensity, demonstrating the specific binding ability of M@P-PDRs to homologous 4T1 cells. (Additional file 1: Fig. S8). Next, the targeting capacity of these M@P-PDR “Nano-targeted cells” to 4T1 cells was evaluated using CLSM. As shown in Fig. 3A, Additional file 1: S9A, and B, 4T1 cells treated with M@P-PDR “Nano-targeted cells” exhibited stronger red fluorescence than that of P-PDR nanospheres, indicating that the cancer cell membrane-coating promoted the intracellular uptake of nanocarriers. Moreover, the red fluorescence enhanced with the extension of coincubation time. This phenomenon was further confirmed by flow cytometry quantitative analyses (Fig. 3B). For instance, after 2 h of incubation, the intracellular uptake rate of the “Nano-targeted cells”-treated group reached 61.67%, while that of the P-PDR nanospheres-treated group was only 12.54%.

The above results indicated that the presence of cancer cell membranes facilitated the intracellular uptake of nanocarriers thus exerting more effective therapeutic effects.

### In vitro therapeutic effects

**Fig. 4:**
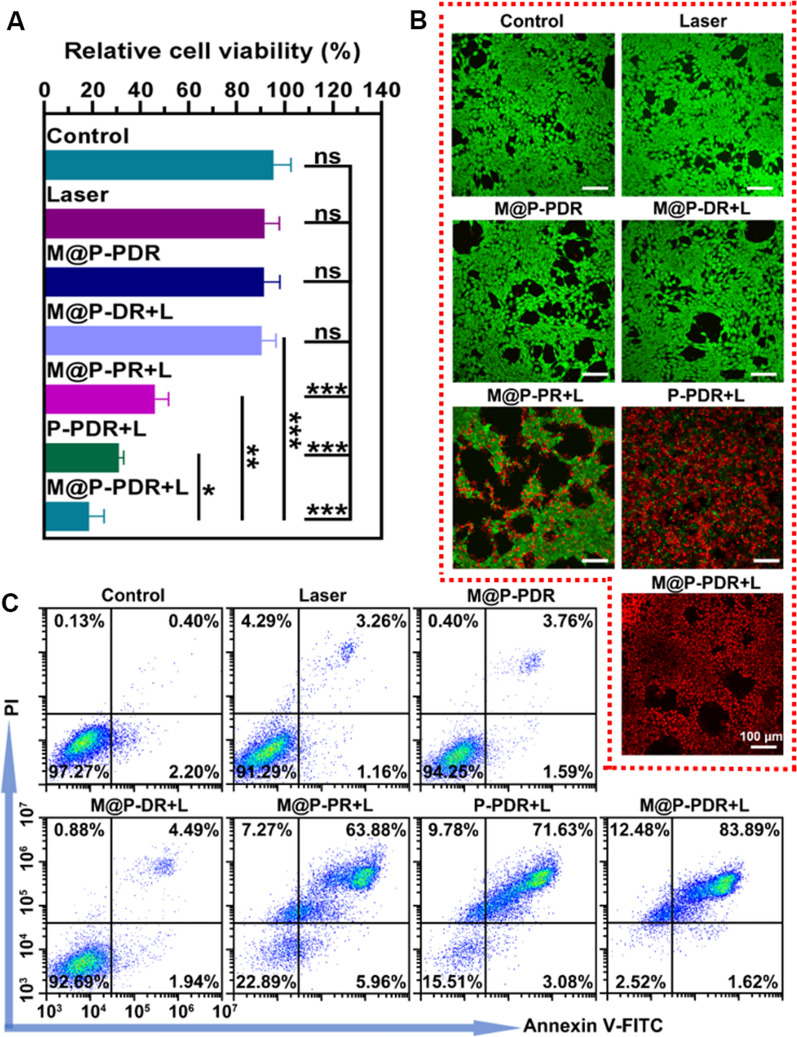
In vitro therapeutic effects of M@P-PDR. **A** CCK8 results after various treatments (n = 3, t-test, *p < 0.05, **p < 0.01, ***p < 0.001, ****p < 0.0001). **B** CLSM images of 4T1 cells co-stained with CAM and PI after various treatments to distinguish the live (green fluorescence) and dead (red fluorescence) cells. **C** Flow cytometry results after various treatments

M@P-PDR nanospheres have been demonstrated to act as PTCAs to convert light energy into thermal energy. The photothermal effects combined with a chemotherapeutic drug (DTX) of M@P-PDR nanospheres against 4T1 cells were evaluated next. According to the results of the CCK-8 assay (Fig. 4A), the cell viability in M@P-PR + L group was 46.14 ± 5.62%, showing the high efficacy of PTT against tumor cells. The M@P-PDR + L group showed a lower cell viability (18.75 ± 6.21%), probably because the released DTX had a certain killing effect on cancer cells. The cell viability of the P-PDR + L group was 30.93 ± 2.11%, which was lower than that of the M@P-PDR + L group, as the cancer cell membrane modification could have promoted more therapeutic agents to accumulate in the tumor cells to mediate the therapeutic processes. The cell viabilities of the laser only group, M@P-PDR only group and the M@P-DR + L group were 91.67 ± 6.08%, 91.26 ± 6.70% and 90.43 ± 5.87%, respectively, which were not significantly different compared with that of the control group (95.30 ± 7.30%). Cell damage was also analyzed by flow cytometry (Fig. 4C), and the results were consistent with the CCK-8 results. Furthermore, cells after various treatments were also stained with CAM/PI to distinguish the live (green fluorescence) and dead (red fluorescence) cells. As shown in Fig. 4B, in the M@P-PDR + L group, almost all cancer cells died, showing bright red fluorescence, while in the P-PDR + L group, some of the cancer cells appeared green due to the lack of efficient intracellular uptake. According to the above results, PTT combined with chemotherapy can inhibit the activity of tumor cells, and the presence of cancer cell membranes optimizes the therapeutic effect of tumors.

### Activation of DCs in vitro

**Fig. 5:**
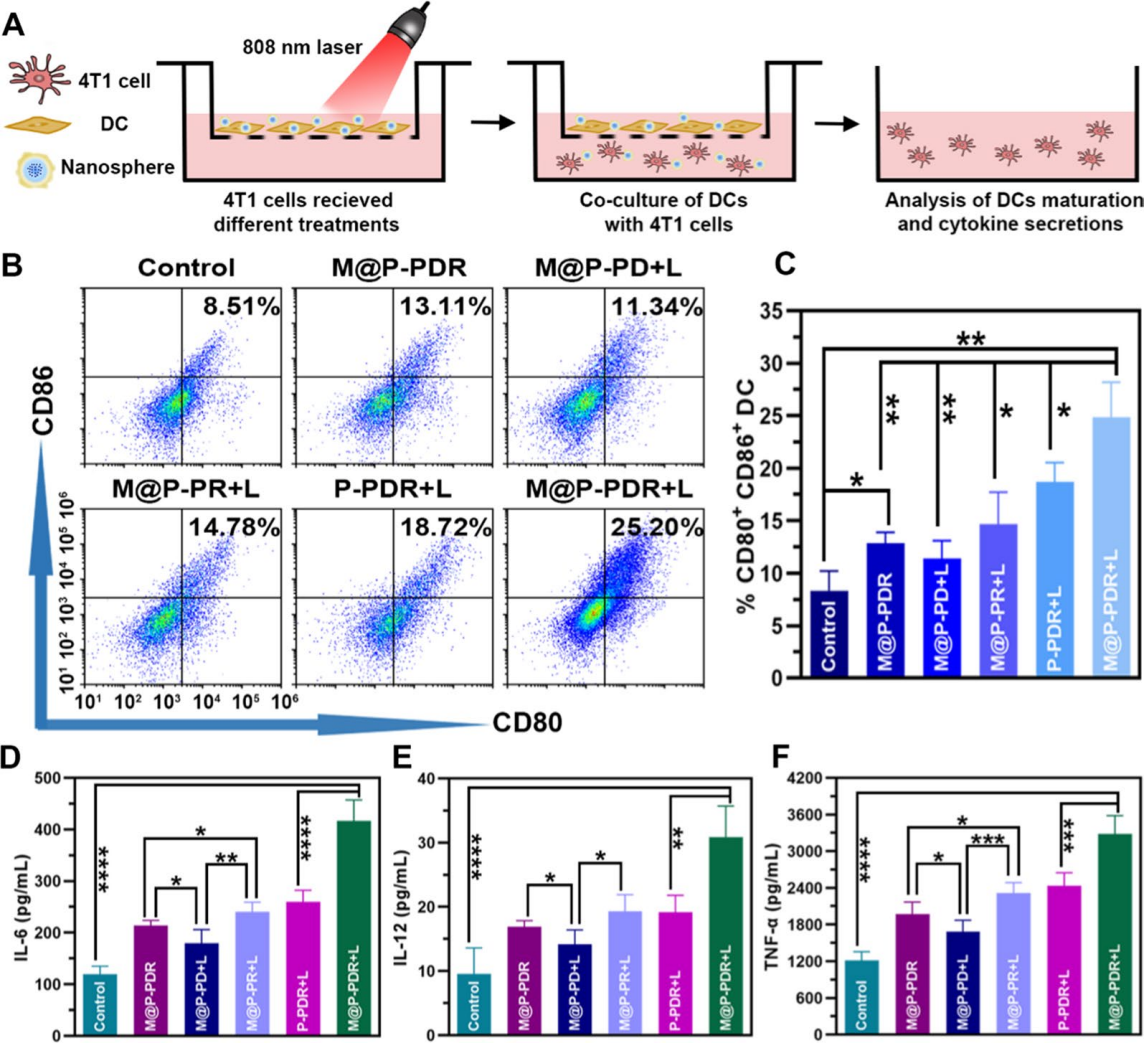
**A** The design scheme of the transwell system experiment. **B**–**C** The expression levels of CD11c^+^, CD86^+^and CD80^+^on the surface of DCs analyzed by flow cytometry after different treatments (n = 3). **D**–**F** The secretion of IL-6, IL-12 and TNF-α in DC suspensions after different treatments (n = 5, t-test, *p < 0.05, **p < 0.01, ***p < 0.001, ****p < 0.0001)

It is well known that dead or dying cells release TAAs and damage-associated molecular patterns (DAMPs), such as heat shock protein 70 (Hsp70) and calreticulin, which can activate antitumor immune responses. These processes are also likely to occur as a result of the abovementioned therapeutic effects. Immune adjuvants such as R837 can further enhance this response. Recognition of R837 by immune cells, such as DCs, that express TLR 7 can significantly promote DC maturation and produce a range of pro-inflammatory cytokines, including TNF-α (a key marker of cellular immune activation), IL-6 and IL-12 (key markers of innate immunity), thereby stimulating T cell responses [22]. DCs, as the most powerful antigen-presenting cells, play an essential role in activating antitumor immune responses [54]. In general, DCs capture TAAs released from dead or dying cancer cells and process them for presentation on major histocompatibility complex class I (MHC-I) molecules. Antigen-loaded DCs then migrate from peripheral tissues to the T cell zone of the draining lymph nodes, where antigen presentation promotes the differentiation of naive T cells into CTLs. Eventually, antigen-stimulated T cells leave the lymph nodes and migrate to metastatic tumors, achieving immunotherapy [55, 56]. Notably, only mature DCs elicit CTLs antitumor responses [57]. The upregulation of typical markers, including costimulatory molecules (CD11c + , CD80 + , CD86 +), indicates the degree of DC maturation. Considering the strong cytotoxic effects induced by M@P-PDR, we investigated whether M@P-PDR-mediated therapy activates immune responses in vitro by using a transwell system in which differently treated 4T1 cancer cells and untreated bone marrow-derived DCs (naive) were seeded in the upper and lower chambers, respectively (Fig. 5A). The maturation efficacy of DCs was measured by flow cytometry (Fig. 5B, C). A slight increase in DC maturation was observed in the M@P-PDR-treated group, which was probably due to the inevitable release of a small amount of R837 from these nanospheres. Compared to the M@P-PD + L group (without R837), the level of DC maturation in the M@P-PDR + L group was greatly increased, which further indicated the role of R837 in promoting the maturation of DCs. Relevant cytokines (TNF-α, IL-6 and IL-12) that would be released by mature DCs were measured by ELISA. It was found that the M@P-PDR group and the M@P-PR + L group showed higher secretion levels than the M@P-PD + L group, which could be attributed to the pivotal role of R837. Compared to the untargeted P-PDR + L group, DCs in the M@P-PDR + L group secreted much more cytokines probably due to homologous targeting capacity mediated by cancer cell membranes (Fig. 5D–F).

### Biodistribution and in vivo MR/PA bimodal imaging

**Fig. 6:**
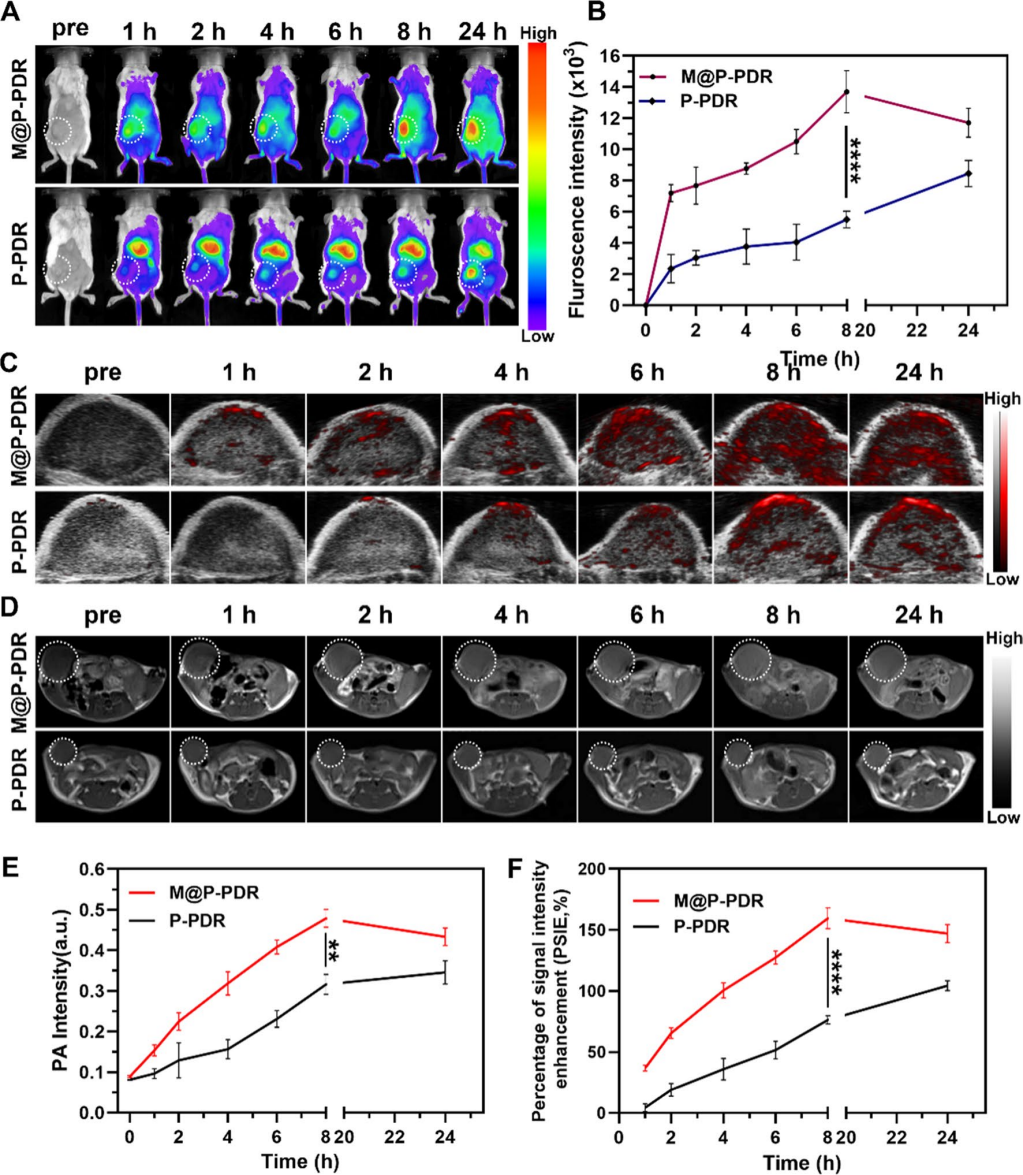
Biodistribution and in vivo MR/PA bimodal imaging of M@P-PDR. **A** Fluorescence images of 4T1 tumor-bearing mice at different time points (pre-injection, 1, 2, 4, 6, 8 and 24 h), and **B** the corresponding fluorescence intensities of tumors (n = 3). **C** In vivo PA images of tumors and **E** the corresponding signal intensities (n = 3). **D** T1-weighted MR images and **F** the corresponding PSIE of tumors (n = 3, t-test, *p < 0.05, **p < 0.01, ***p < 0.001, ****p < 0.0001)

To monitor the biodistribution and in vivo targeting behavior of these M@P-PDR “Nano-targeted cells”, fluorescence imaging of tumor-bearing mice was performed. DiR-labeled M@P-PDR and P-PDR nanospheres were intravenously injected, respectively. In the M@P-PDR-treated group, obvious fluorescence signals at the tumor sites were observed. The signals increased with injection time and reached a peak at 8 h (Fig. 6A, B). The mean fluorescence intensity of the tumors was 13.70 ± 1.35 × 10^3^, which was 2.49-fold higher than that of the P-PDR-treated group (5.50 ± 0.54 × 10^3^), which could result from the homologous targeting capacity of cancer cell membranes. More importantly, significant fluorescence signals were still clearly visible at 24 h postinjection, indicating long-term retention of these M@P-PDR “Nano-targeted cells”. Afterward, tumors and major organs (heart, liver, spleen, lung, kidney) were dissected for ex vivo fluorescence imaging. The fluorescent signals of tumors in the M@P-PDR group were evidently stronger than those of the P-PDR group (p < 0.05) (Additional file 1: Fig. S10A and B). These results clearly indicated that the cancer cell membrane-coated nanospheres were endowed with superior active targeting ability, showing promising possibilities for in vivo precise imaging and effective treatment.

After intravenous injection, small nanoparticles may be cleared by the kidneys, such as the graphene nanomaterials (< 50 nm) designed by Omid Akhavan et al*.* and Zhuang Liu et al*.* [58, 59], whereas larger nanoparticles (> 200 nm) could be recognized and cleared by the RES of liver and spleen [60, 61]. The nanoparticles we synthesized are larger in size (> 200 nm), so they are expected to be cleared by the RES, which may lead to more aggregation of nanoparticles in the liver than that in the kidney. In addition, after intravenous administration, the exogenous nanoparticles would be nonspecifically intercepted by the liver, which is rich in phagocytic cells, resulting in substantial aggregation of nanoparticles in the liver. As shown in Additional file 1: Fig. S10A and B, the M@P-PDR group had fewer nanoparticles aggregated in the liver, which should be ascribed to the reduced clearance of the RES due to the presence of the antigen retention of the cancer cell membrane coating.

The aforementioned in vitro experiments confirmed that M@P-PDR nanospheres could act as contrast agents to enhance both PA imaging and T1-weighted MR imaging. Therefore, bimodal PA and MR imaging performance were further investigated in vivo. As expected, in the M@P-PDR group, the PA signals within tumor regions gradually increased with prolonged time, and reached a peak at 8 h postinjection (0.479 ± 0.022) in comparison with those at preinjection (0.089 ± 0.003) (Fig. 6C, E, Additional file 1: S10C). At 24 h postinjection, the PA signal intensities (0.433 ± 0.022) slightly decreased due to the gradual clearance of these nanospheres from tumor tissues. In contrast, in the P-PDR group without homologous targeting, the PA signals were significantly weaker throughout the time course of the observation. The T1-weighted MR imaging results showed that the tumors in the M@P-PDR group were clearly demarcated from the surrounding normal tissues with clear anatomical structures.

In addition, obvious bright enhancements were observed at the tumor areas over time, reached a peak at 8 h postinjection, and were sustained for 24 h (Fig. 6D, F). PSII was used for quantitative analysis of T1-weighted MR imaging enhancement. Specifically, the average T1-weighted signal intensities in the M@P-PDR group increased by 159.632 ± 8.549% at 8 h postinjection, whereas only 76.784 ± 3.346% enhancement rate was observed in the P-PDR group. The pseudocolor images also clearly showed enhancements (Additional file 1: Figure S10D). The trend of MR imaging is consistent with that of PA imaging and the enrichment was reflected by in vivo fluorescence imaging. These results indicated that the surface modification of cancer cell membranes on P-PDR structures contributed to the efficient accumulation of nanocarriers in tumor sites. Additionally, the excellent PA/MR bimodal imaging performance of these M@P-PDR “Nano-targeted cells” can provide a therapeutic time window and guide NIR laser irradiation, achieving more precise therapy delivery.

### In vivo cocktail therapy evaluation

#### In vivo photothermal performance

**Fig. 7:**
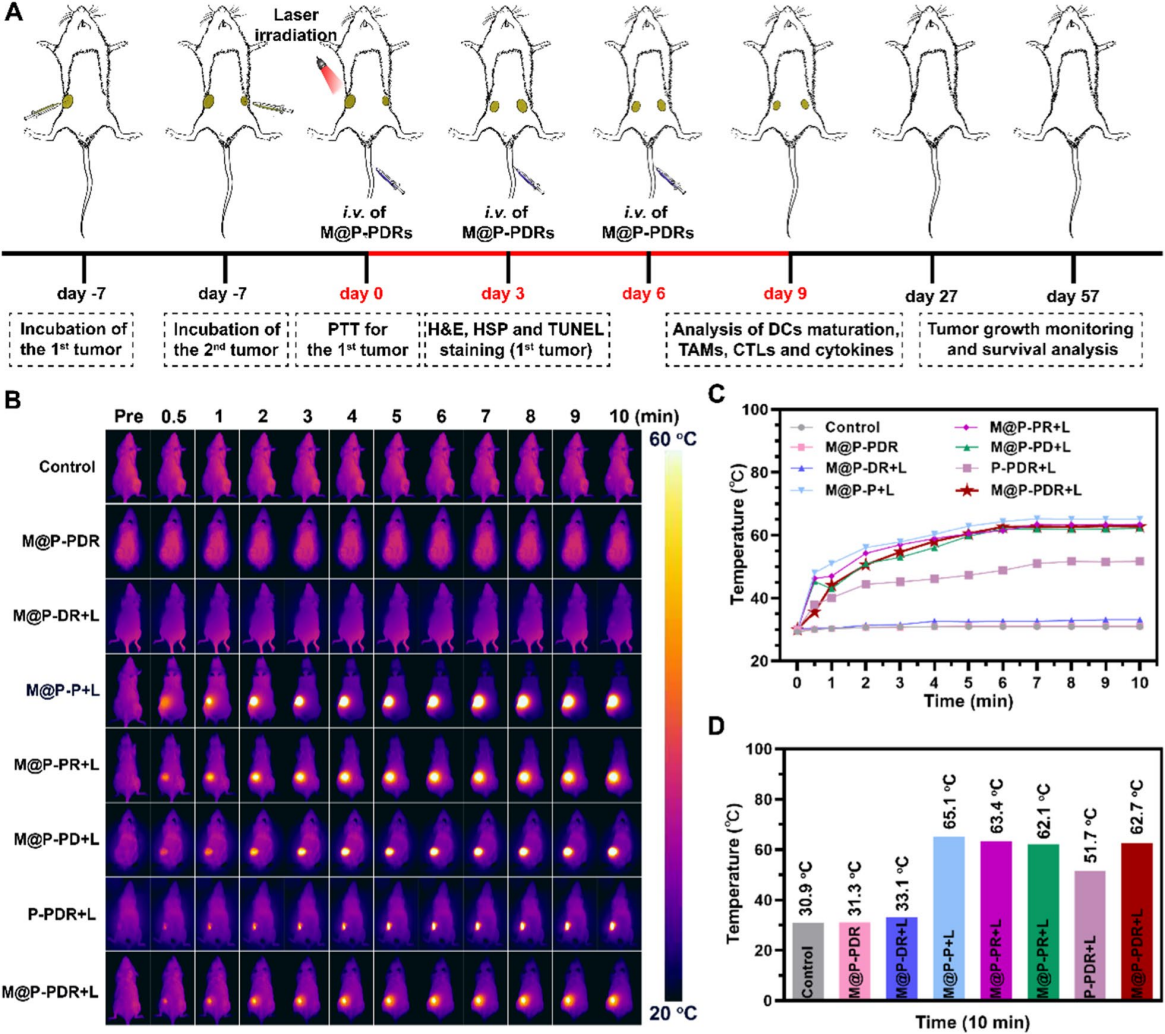
In vivo photothermal performance of the M@P-PDR “Nano-targeted cells”. **A** Schematic illustration of the in vivo experimental design. **B** Infrared thermal images of 4T1 tumor-bearing Balb/c mice under different treatment groups. **C** Photothermal temperature–time curves of the eight groups under laser irradiation. and **D** of the corresponding temperature changes at tumor sites during irradiation

After the enrichment of the PTCAs in tumor areas, the local temperature would rise under laser irradiation. The tumors were exposed to laser irradiation 8 h after the intravenous injection of nanospheres, and the temperature changes were monitored by an infrared thermal imaging system. As shown in Fig. 7B–D, the temperatures of tumors presented a slight increase in the M@P-PDR and M@P-DR + L groups compared to the control group in the absence of laser or PB nanoparticles. A significant temperature increase was observed in the groups with concurrent laser irradiation and PB components, demonstrating excellent in vivo photothermal performance. The temperature in the M@P-PDR + L group increased to 62.7 ℃, which was much higher than that of the P-PDR + L group (51.7 ℃) without homologous targeting capacity. Assisted by cancer cell membrane coating, PTCAs could accumulate in tumors more efficiently to achieve more efficient and uniform localized hyperthermia.

#### Immune responses evaluation

**Fig. 8:**
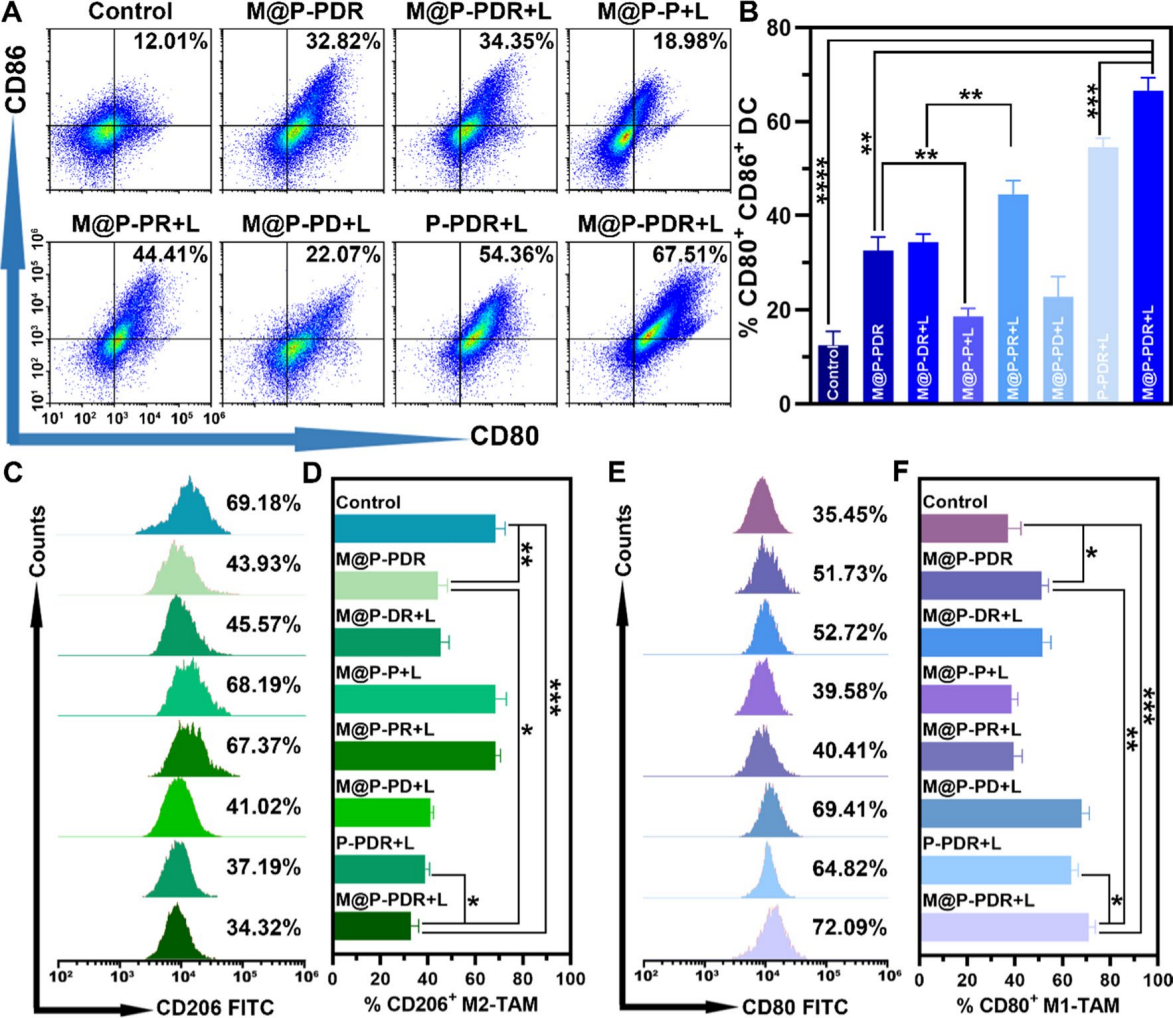
Degree of in vivo DCs maturation and polarization of TAMs based on M@P-PDR “Nano-targeted cells”. **A** Flow cytometric analysis of DCs maturation in primary tumors (1st) of mice in different treatment groups. and **B** the corresponding quantification of DCs maturation (n = 3). **C** Flow cytometric analysis of M2-TAMs (CD206^+^ F4/80^+^ CD11b^+^) in primary tumors (1st) and **D** the corresponding quantification of M2-TAMs (n = 3). **E** Flow cytometric analysis of M1-TAMs (CD80^+^ F4/80^+^ CD11b^+^) in primary tumors (1st) and **F** the corresponding quantification of M1-TAMs (n = 3, t-test, *p < 0.05, **p < 0.01, ***p < 0.001, ****p < 0.0001)

Encouraged by the activation of DCs in vitro, the in vivo immune responses were evaluated next. The experimental design is shown in Fig. 7A. Tumors were inoculated at both the left and right mammary fat pads of mice in chronological order, and set as primary tumor (1st) and artificial mimicked metastasis (2nd), respectively. The mice were randomly divided into eight groups and administered different treatments. The day when the treatments designated was set as Day 0. To analyze the DC maturation level in vivo, primary tumors (1st) (Fig. 8A, B), metastatic tumorS (2nd) (Additional file 1: Fig. S11A, B) and lymph node (Additional file 1: Fig. S11A, C) were collected to make single-cell suspensions for flow cytometry assay on day 9. Similar to the in vitro results, the integration of the R837 immune adjuvant endowed M@P-PDR “Nano-targeted cells” with a much stronger ability to promote DC maturation, accompanied by increased cytokine secretion in vivo. In detail, the M@P-PDR + L group induced the highest level of DC maturation (66.56 ± 2.78%), which was significantly higher than the M@P-PD + L group without R837 (22.81 ± 4.26%), M@P-PDR “Nano-targeted cells” alone (32.65 ± 2.84%), and P-PDR + L group without homologous targeting capability (54.55 ± 1.96%). After PTT combined with chemotherapy, tumor tissues were damaged, and tumor cell fragments released TAAs, showing an “autologous cancer vaccine-like” function [62]. Especially in the presence of immune adjuvants, it can promote the maturation of DCs more efficiently [63].

Although activation can be established through multiple pathways, the immunosuppressed TME often results in a suboptimal immune response. As an important component of the TME, TAMs play an important role in tumor immune regulation. As a major member of the TME, M2-phenotype macrophages promote tumor cell invasion, and metastasis and suppress immune responses by secreting relevant cytokines (*e.g.*, IL-10), M1-phenotype macrophages counteract tumor growth, and promote inflammatory and immune responses by secreting relevant cytokines (*e.g.*, IL-6, IL-12 and TNF-α) [64–66]. In a pro-immune response pathway, M2-phenotype cancer-promoting TAMs can be repolarized to M1-phenotype cancer-suppressing TAMs under certain conditions. In this study, DTX was introduced to promote the polarization of M1-phenotype to M2-phenotype TAMs. To analyze the polarization of M2-phenotype macrophages, we studied the presence of M1 (F4/80^+^ CD11b^+^ CD80^+^) and M2 (F480^+^ CD11b^+^ CD206^+^) markers on Day 9 after various treatments. As shown in Fig. 8C–F and Additional file 1: S12A, the expression of F4/80^+^ CD11b^+^ CD80^+^ was significantly upregulated in the groups integrated with DTX, accomplished by the downregulation of F480^+^ CD11b^+^ CD206^+^ expression, which demonstrated the potency of DTX to promote the polarization of M1-phenotype to M2-phenotype TAMs.

**Fig. 9:**
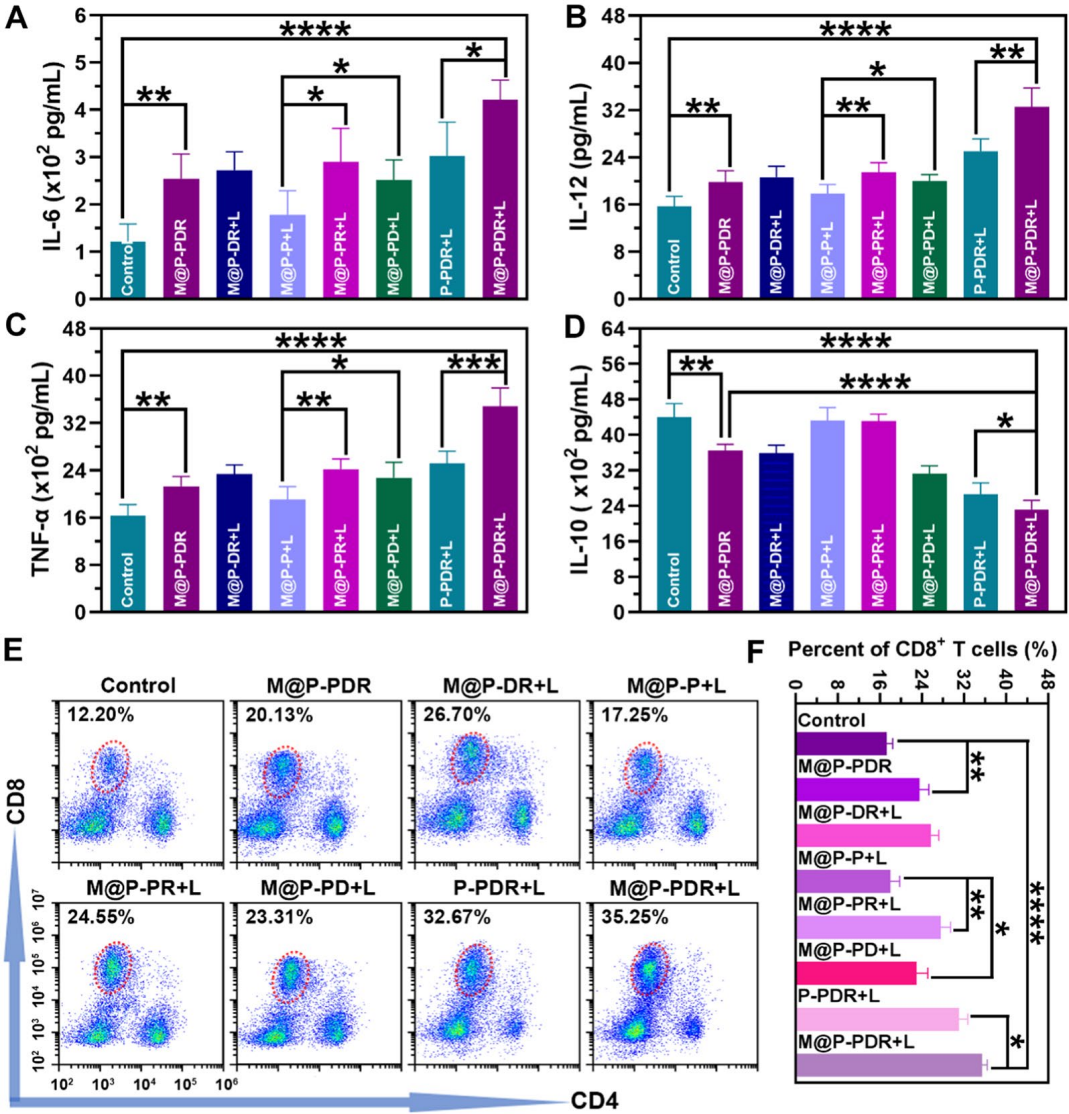
In vivo immunostimulatory effects based on M@P-PDR “Nano-targeted cells”. **A**–**D** The secretion levels of IL-6, IL-12, TNF-α and IL-10 measured by ELISA assay (n = 5). **E** Flow cytometric analysis of CD8^+^ T cell in the spleens of mice in different groups. **F** Quantification of CD8^+^ T cells (n = 3, t-test, *p < 0.05, **p < 0.01, ***p < 0.001, ****p < 0.0001)

When the host immune status changes, the levels of cytokines in vivo will change correspondingly. Here, the levels of IL-6, IL-12, TNF-α and IL-10 in the eight groups were investigated by ELISA on Day 9. As shown in Fig. 9A–C, the levels of these cytokines were consistent with the change in the host immune status (DC maturation and polarization of TAMs) discussed before. The groups integrated with DTX downregulated the production of IL-10 (Fig. 9D), further demonstrating that DTX has an excellent ability to promote the polarization of M1-phenotype to M2-phenotype TAMs.

CD8^+^ T cells, namely CTLs, are essential for the anticancer immune response. To evaluate the T cell response in vivo, the spleens of mice were collected on Day 9 and T cells in the spleens were analyzed using flow cytometry. The results (Fig. 9E, F) showed that the infiltration of CD8^+^ T cells in the M@P-PDR + L group was 35.50 ± 0.96%, which was significantly higher than that in the control group (17.33 ± 1.13%), the M@P-PDR group (23.54 ± 1.83%), the M@P-DR + L group (25.70 ± 1.57%), the M@P-P + L group (18.01 ± 1.77%), the M@P-PR + L group (27.64 ± 1.86%), the M@P-PD + L group (22.99 ± 2.18%), and the P-PDR + L group (31.09 ± 1.71%), indicating that the processes (PTT, chemotherapy, DC maturation and polarization of TAMs) mediated by M@P-PDR “Nano-targeted cells” triggered excellent antitumor immune responses. Consistently, immunofluorescence images of the primary and metastatic tumors also revealed substantial infiltration of CD8^+^ T cells (Additional file 1: Fig. S12B).

#### In vivo antitumor therapy for primary and metastatic tumors

**Fig. 10:**
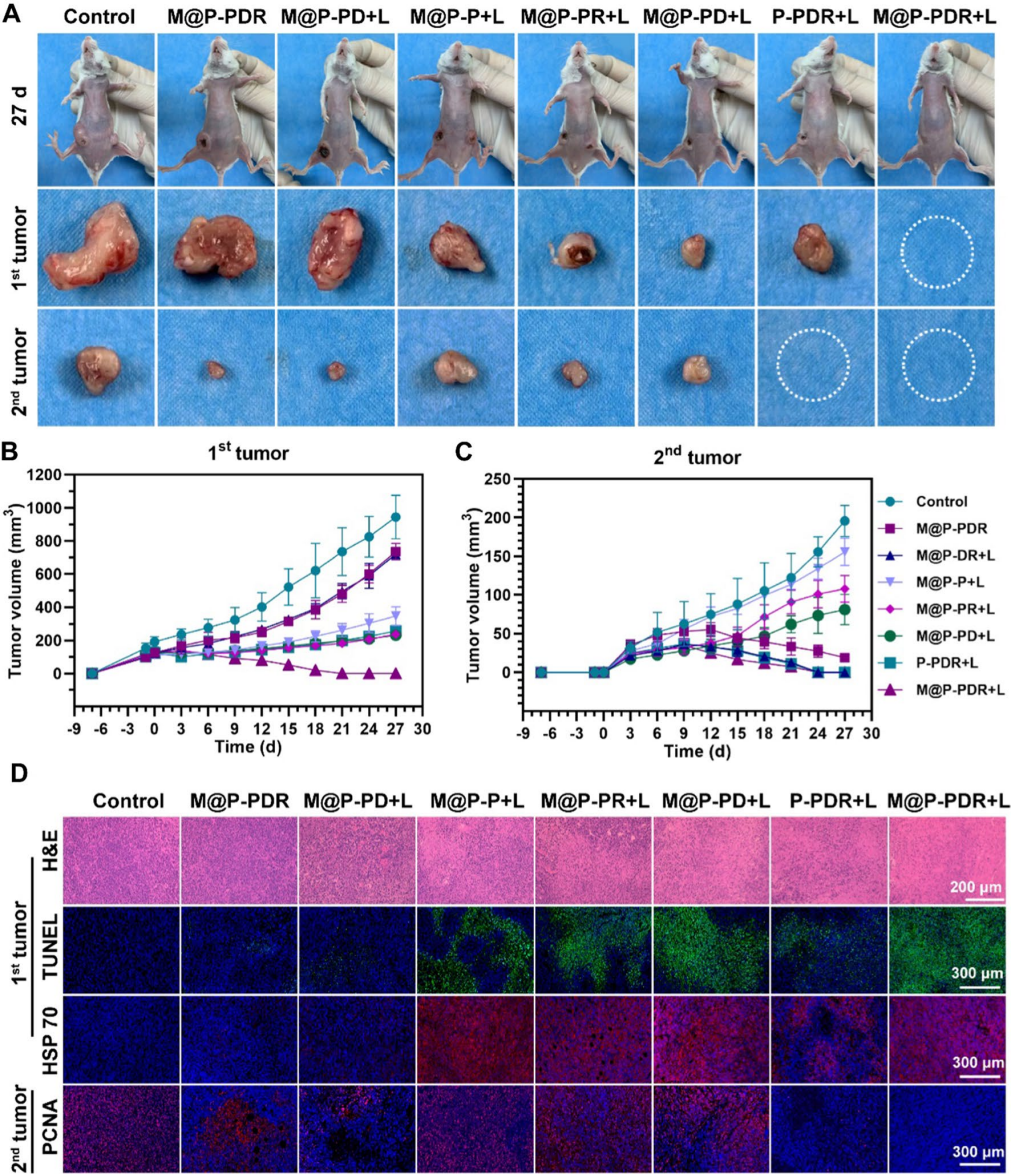
Anti-tumor effects of cocktail therapy based on M@P-PDR “Nano-targeted cells”. **A** Digital photos of 4T1 tumors on both sides in vivo and ex vivo on day 27 after different treatments. **B** Growth curves of the primary tumors (1st) and **C** the distant tumors (2nd) in different groups (n = 5, t-test, *p < 0.05, **p < 0.01, ***p < 0.001, ****p < 0.0001). **D** H&E staining, TUNEL staining and HSP70 staining images of the primary tumor (1st) excised at day 3 after different treatments, and PCNA staining images of the distant tumor (2nd) excised at day 9 after different treatments

Encouraged by the satisfactory immune response, we believe that the M@P-PDR-based cocktail therapy could be a promising candidate to combat distant tumors. In the following study, we investigated whether such a strong immune response initiated by M@P-PDR was available for long-term inhibition of metastatic tumors. The therapeutic efficacy was evaluated by monitoring the growth of primary tumors and distant tumors. Compared with the primary tumors (1st) in the control group, all other treated groups exhibited certain inhibitary effects on tumor growth (Fig. 10A, B, Additional file 1: S13, S14A). In detail, no significant difference was found between the M@P-PDR group and the M@P-DR + L group, whereas limited tumor growth regression occurred as a result of the release of DTX and R837 in tumor sites. However, the photothermal effect of M@P-P greatly inhibited tumor progression, with a 3.05-fold increase in comparison to the primary tumor volume. In particular, the primary tumors in the M@P-PDR + L group were remarkably inhibited, suggesting the excellent antitumor efficiency of such cocktail therapy that concurrently integrates PTT, chemotherapy and immunotherapy. For distant tumor growth in Fig. 10A, B, Additional file 1: S13 and S14B, the tumors in the M@P-PDR + L group were also effectively inhibited, which could be ascribed to the strong immune response resulting from R837-induced DC maturation (immune activation) and the DTX-mediated polarization of TAMs (relief of immunosuppression). To compare the therapeutic effects more directly, the tumors in each group were collected at 3 d postinjection for TUNEL and HSP70 staining (Fig. 10D). The results showed that, in the presence of both PTCAs and laser irradiation, the expression of HSP70 was higher than that in other groups, presenting obvious red fluorescence. The M@P-PDR + L group showed a higher expression of HSP70 than the P-PDR + L group, indicating the specific targeting effect of cancer cell membranes on the accumulation of nanotherapeutic agents in tumor sites. H&E and TUNEL staining presented a similar tendency in which the photothermal effects induced massive tumor necrosis. The PCNA results of distant tumors revealed that the M@P-PDR + L group exerted extensive antitumor effects, with negligible tumor proliferation. In addition to the pathological examination, the survival rates of mice in each group were monitored until Day 57 (Additional file 1: Fig. S14C). The mice in the M@P-PDR + L group survived without obvious tumor recurrence. These results confirmed that the powerful systemic immune response of M@P-PDR-based cocktail therapy effectively inhibited the growth of distant tumors, providing a new strategy for PTT/chemotherapy/immune therapy. H&E staining of major organs (Additional file 1: Fig. S15) and the negligible body weight changes (Additional file 1: Fig. S16) further demonstrated the satisfactory biosafety of this synergistic therapeutic modality.

Here, a comparison with previously published tumor-therapeutic modalities containing those entities summarized into a table showing the advantages of this new cocktail therapeutic strategies (Additional file 1: Table S1). As one of the traditional cancer treatment modalities, chemotherapy can inhibit tumor growth to some extent [67, 68]. However, most chemotherapeutic drugs have the barriers of dose-dependent toxicity and unsatisfactory tumor accumulation, which significantly hindered its application [69]. Some studies have demonstrated that DTX can effectively reverse the immunosuppressive TME by polarizing protumoral M2-phenotype TAMs to tumoricidal M1-phenotype TAMs [24]. Immunotherapy has received increasing attention due to its ability to activate host defenses to identify, attack, and eradicate cancer cells [70, 71]. However, low immune response rate and individual differences undermine its antitumor efficacy. Moreover, high cost and the existence of immune-related adverse reactions (IrAEs) further limit its application [4]. PTT is widely used for cancer treatment because of its noninvasiveness, low energy consumption, and minimal toxicity to normal tissues [72, 73]. However, limited light penetration depth may lead to incomplete tumor ablation can cause tumor recurrence or even metastasis. Although PTT can activate an immune response that inhibits recurrence and metastasis, the immunosuppressive TME, including myeloid-derived suppressor cells (MDSCs), prostate cancer M2-TAMs and regulatory T cells (Tregs), leads to ineffective antitumor immune response and immunotherapeutic resistance [4, 74]. In this study, R837 and DTX was further integrated into PTT to enhance the immune response and relieve the immunosuppressive TME. Cocktail therapy not only effectively ablates the primary tumor, but also reverses the immunosuppressive TME and enhances the antitumor efficacy.

## Conclusion

In summary, we rationally proposed a “Nano-targeted cells”-based cocktail therapy, in which PTT was combined with DTX-enhanced immunotherapy, creating a “doomsday storm” for tumors. These as-synthesized “Nano-targeted cells” actively accumulate at tumor sites due to their homologous targeting capability, which can be guided by PA/MR bimodal imaging. Upon laser irradiation, PTT will be triggered, and TAAs will be subsequently released. The released TAAs, together with the immune adjuvant R837, drive the maturation of DCs, secreting cytokines, including TNF-α, IL-6 and IL-12. Furthermore, the chemotherapeutic drug DTX polarizes protumoral M2-phenotype oncogenic TAMs to tumoricidal M1-phenotype oncogenic TAMs, relieving the immunosuppressive TME, accompanied by a decrease in IL-10. The above processes promote the infiltration of CTLs for treating distant metastasis. Primary tumors and metastasis are significantly inhibited. “Nano-targeted cells”-based therapeutic cocktail therapy is a promising approach to promote tumor regression and counter metastasis/recurrence.

## Supplementary Information

**Additional file 1: Fig. S1**. Image of PB nanoparticles. **Fig. S2**. FTIR spectra of PB nanoparticles, drugs (R837 and DTX), PLGA nanospheres, P-P nanospheres, P-PDR nanospheres and M@P-PDR nanospheres. **Fig. S3**. Standard curve of PB, R837 and DTX. **Fig. S4**. PA imaging intensities under full-spectrum scanning. **Fig. S5**. Cell viabilities of 4T1 cells after co-incubation with of M@P-PDR and P-PDR for 12 h. **Fig. S6**. In vivo biosafety of M@P-PDR. **Fig. S7**. Western blotting analysis of membrane-specific protein markers. **Fig. S8**. Validation Homologous Targeting Capability of M@P-PDR. (A) CLSM images of 4T1 cells, MDA-MB-231 cells or SKBR3 cells coincubated with M@P-PDR for 2h, respectively. (B) The corresponding flow cytometry quantitative analyses of intracellular uptake. **Fig. S9**. Confocal microscopy images of 4T1 cells treated with P-PDR and M@P-PDR. **Fig. S10**. (A) Fluorescence images of major organs and tumors in M@P-PDR and P-PDR treated groups 24 h after intravenous administration and (B) the corresponding fluorescence intensities. (C) In vivo PA images of tumors. (D) T1-weighted MR images of 4T1 tumor-bearing mice. **Fig. S11**. (A) Flow cytometric analysis of DCs maturation in distant tumors (2nd) and lymph nodes. (B) The corresponding quantification of DCs maturation in distant tumors (2nd) and (C) the corresponding quantification analysis of DCs maturation in lymph nodes (LNs). **Fig. S12**. (A) Immunofluorescence images of CD80 and CD206 in distant tumors (2nd) on day 9. (B) Immunofluorescence images of CD8+ T cells in the distant tumors (2nd) on day 9 after different treatments. **Fig. S13**. Images of mice during the 27-day treatment period. **Fig. S14**. (A) Statistical analysis of primary tumors (1st) and (B) distant tumors (2nd) on day 27. (C) Time-dependent body weight curves of mice. (D) Survival curves of mice after different treatments. **Fig. S15**. H&E staining of the major organs of all groups collected on day 3 after different treatments. **Fig. S16**. Time-dependent body weight curves of mice.** Table S1**. The comparison of photothermal/chemo-/immuno- cocktail therapy with other tumor therapeutic modalities

## Data Availability

All data analyzed during this study are included in this published article.
