# Supplementary material for: Cancer cell membrane-coated nanoparticles for bimodal imaging-guided photothermal therapy and docetaxel-enhanced immunotherapy against cancer
Source: J Nanobiotechnology. 2021 Dec 24;19:449. doi: 10.1186/s12951-021-01202-x (PMC8710014; doi:10.1186/s12951-021-01202-x)
Supplement: Supplementary file 1 — Additional file 1: Fig. S1. Image of PB nanoparticles. Fig. S2. FTIR spectra of PB nanoparticles, drugs (R837 and DTX), PLGA nanospheres, P-P nanospheres, P-PDR nanospheres and M@P-PDR nanospheres. Fig. S3. Standard curve of PB, R837 and DTX. Fig. S4. PA imaging intensities under full-spectrum scanning. Fig. S5. Cell viabilities of 4T1 cells after co-incubation with of M@P-PDR and P-PDR for 12 h. Fig. S6. In vivo biosafety of M@P-PDR. Fig. S7. Western blotting analysis of membrane-specific protein markers. Fig. S8. Validation Homologous Targeting Capability of M@P-PDR. (A) CLSM images of 4T1 cells, MDA-MB-231 cells or SKBR3 cells coincubated with M@P-PDR for 2h, respectively. (B) The corresponding flow cytometry quantitative analyses of intracellular uptake. Fig. S9. Confocal microscopy images of 4T1 cells treated with P-PDR and M@P-PDR. Fig. S10. (A) Fluorescence images of major organs and tumors in M@P-PDR and P-PDR treated groups 24 h after intravenous administration and (B) the corresponding fluorescence intensities. (C) In vivo PA images of tumors. (D) T1-weighted MR images of 4T1 tumor-bearing mice. Fig. S11. (A) Flow cytometric analysis of DCs maturation in distant tumors (2nd) and lymph nodes. (B) The corresponding quantification of DCs maturation in distant tumors (2nd) and (C) the corresponding quantification analysis of DCs maturation in lymph nodes (LNs). Fig. S12. (A) Immunofluorescence images of CD80 and CD206 in distant tumors (2nd) on day 9. (B) Immunofluorescence images of CD8+ T cells in the distant tumors (2nd) on day 9 after different treatments. Fig. S13. Images of mice during the 27-day treatment period. Fig. S14. (A) Statistical analysis of primary tumors (1st) and (B) distant tumors (2nd) on day 27. (C) Time-dependent body weight curves of mice. (D) Survival curves of mice after different treatments. Fig. S15. H&E staining of the major organs of all groups collected on day 3 after different treatments. Fig. S16. Time-dependent body weight cur [file 12951_2021_1202_MOESM1_ESM.docx]

***Supplementary Materials for***

**Cancer Cell Membrane-Coated Nanoparticles for Bimodal Imaging-Guided Photothermal Therapy and Docetaxel-Enhanced Immunotherapy against Cancer**

Qiaoqi Chen^1†^, Liang Zhang^1, 2†^, Lin Li^1^, Mixiao Tan^1^, Weiwei Liu^1^, Shuling Liu^3^, Zhuoyan Xie^4^, Wei Zhang^1^, ZhigangWang^1^, Yang Cao^1^, Tingting Shang^1*^, Haitao Ran^1*^

^1^Chongqing Key Laboratory of Ultrasound Molecular Imaging, Institute of Ultrasound Imaging, The Second Affiliated Hospital, Chongqing Medical University, Chongqing 400010, P. R. China;

^2^Department of Ultrasound, The First Affiliated Hospital, Chongqing Medical University, Chongqing 400042, P. R. China;

^3^Department of Radiology, Chongqing University Cancer Hospital & Chongqing Cancer Institute & Chongqing Cancer Hospital, Chongqing 400030, P. R. China;

^4^Chongqing General Hospital, University of Chinese Academy of Sciences, Chongqing 401121, P. R. China.

^†^ Qiaoqi Chen and Liang Zhang contributed equally to this work.

^*^ Correspondence: stt419@hospital.cqmu.edu.cn; ranhaitao@hospital.cqmu.edu.cn.

**Synthesis of PB Nanoparticles**

PB nanoparticles were prepared according to the previous literatures [1]. Briefly, 0.5 mmol citric acid was added to FeCl_3_ (20 mL, 1.0 mM) aqueous solution. The mixture was then stirred to fully mix and dissolve the monomers. The mixture was added dropwise to K4[Fe(CN)_6_] (20 mL, 1.0 mM) aqueous solution containing the same amount of citric acid with continuous magnetic stirring at a temperature of 60°C. Transparent blue solutions were obtained, and stirring was continued until it cooled to room temperature. PB nanoparticles sediments were obtained after a centrifugation process (500,000 ×g, 60 min). Finally, the PB nanoparticles dissolved in double-distilled water were dialyzed with a 14,000 MWCO membrane for 24 h to remove unbound citric acid molecules and other small ions and stored at 4 °C for future use [2].


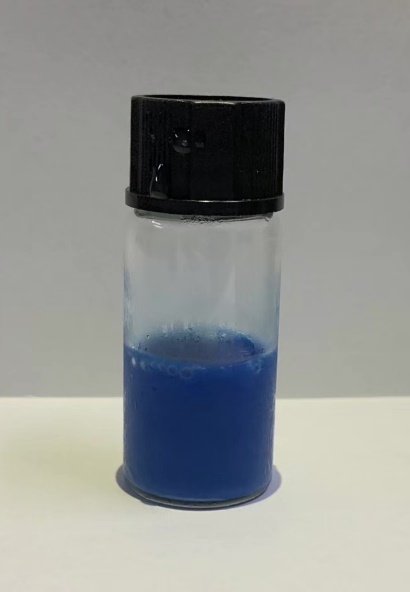


**Fig. S1.** Image of PB nanoparticles dispersed in water.


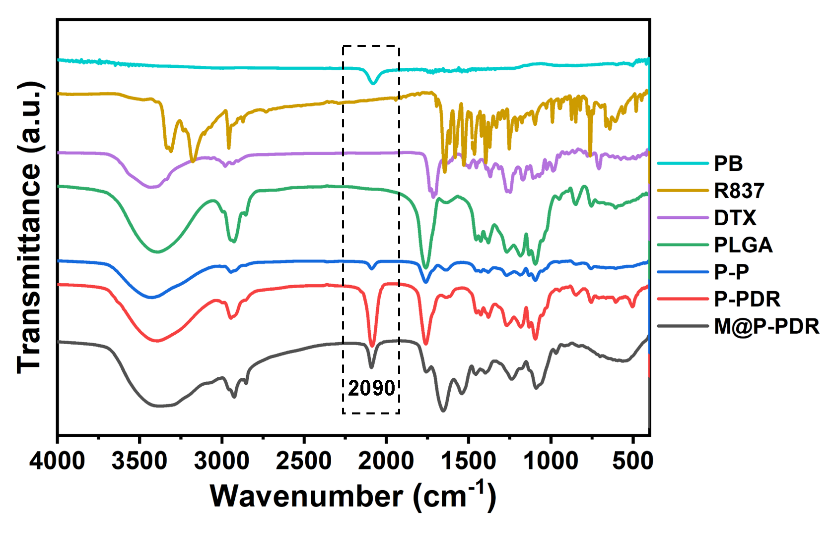


**Fig. S2.** FTIR spectra of PB nanoparticles, drugs (R837 and DTX), PLGA nanospheres, P-P nanospheres, P-PDR nanospheres and M@P-PDR nanospheres.


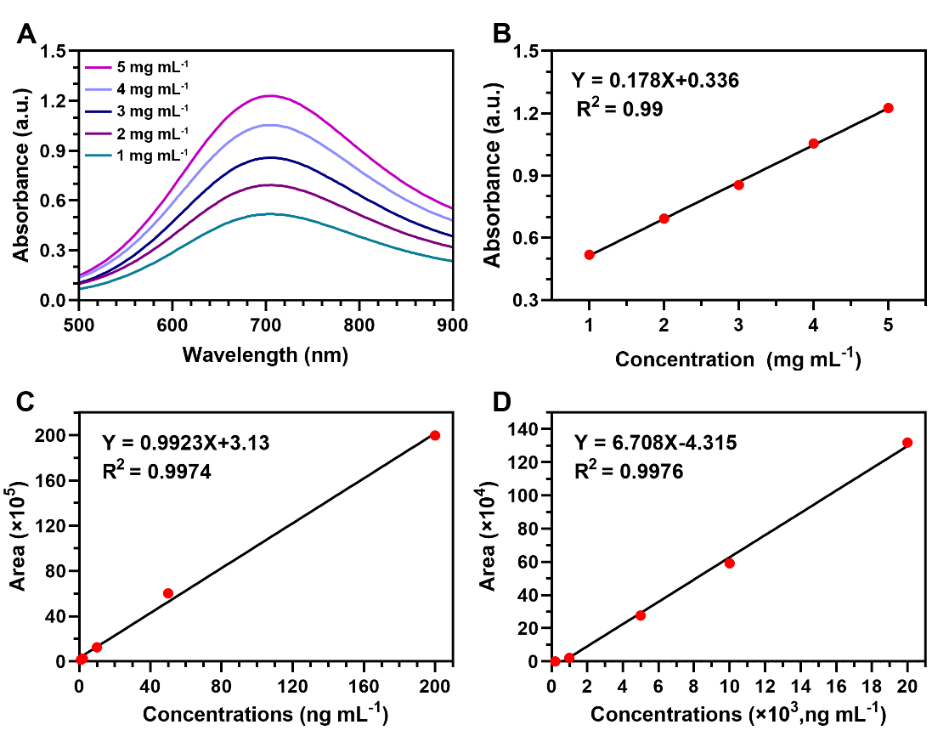


**Fig. S3.** Characterization of the M@P-PDR. (A) UV–vis–NIR absorbance spectra of PB nanoparticles at different concentrations. (B) The relative absorbance intensity of PB nanoparticlesC in UV–vis–NIR spectrum at the wavelength of 700 nm. (C) Standard curve of R837 based on LC-MS. (D) Standard curve of DTX based on LC-MS.


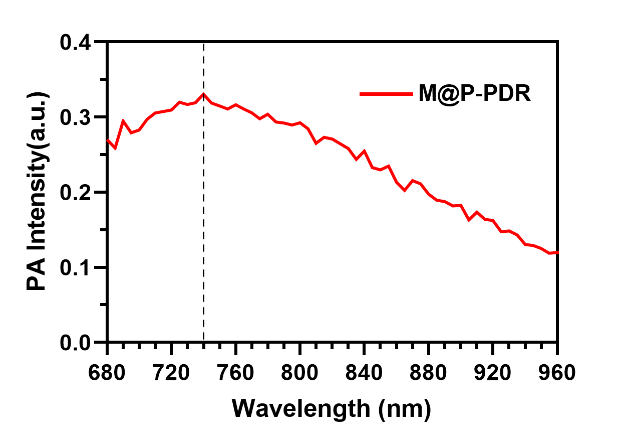


**Fig. S4.** PA imaging intensities under full-spectrum scanning (ranging from 680 to 960 nm).


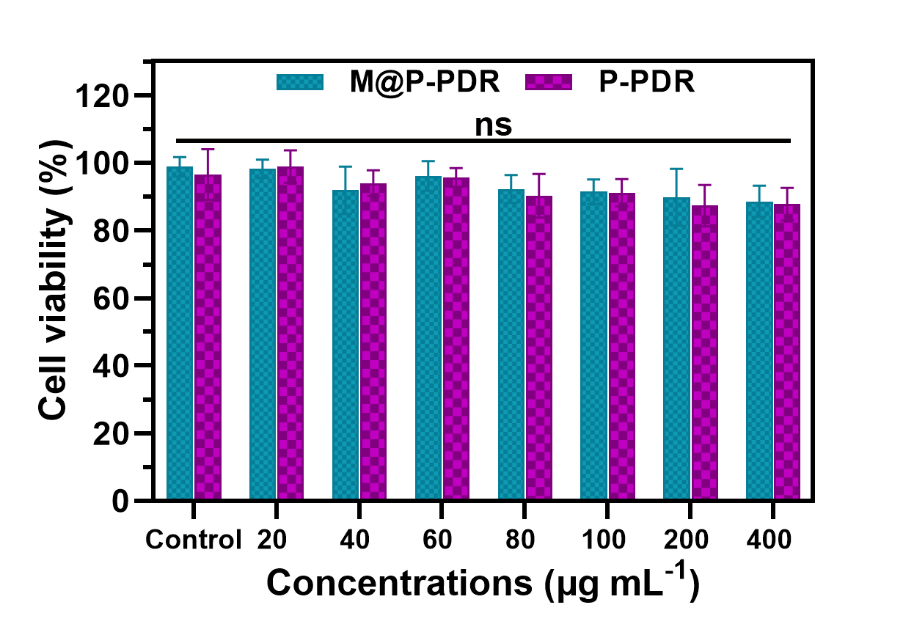


**Fig. S5.** Cell viabilities of 4T1 cells after co-incubation with various concentrations (0, 20, 40, 60, 80, 100, 200 and 400 µg mL^-1^) of M@P-PDR and P-PDR for 24 h (n = 3, t-test, *p < 0.05, **p < 0.01, ***p < 0.001, ****p < 0.0001).


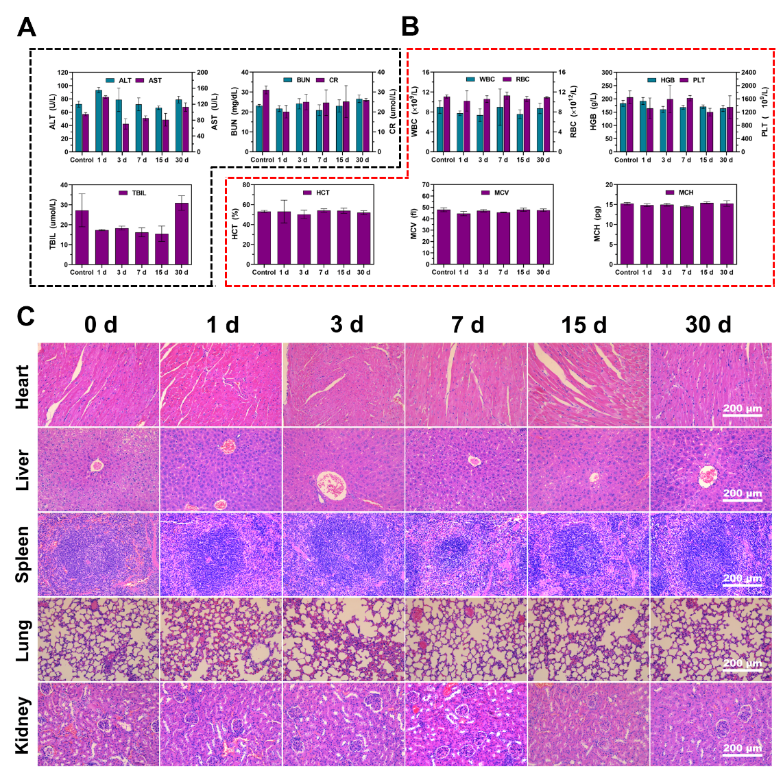


**Fig. S6.** *In vivo* biosafety of M@P-PDR. (A) Serum biochemical data including: alanine aminotransferase (ALT), glutamic aminotransferase (AST), total bilirubin (TBIL), blood urea nitrogen (BUN) and creatinine (CR) obtained from healthy BALB/c mice sacrificed after 1 d, 3 d, 7 d, 15 d and 30 d of intravenous injection of M@P-PDR (3 mg mL^-1^, 200 μL per mouse) and measured by hematological analysis. (B) Complete blood count, including: white blood cells (WBC), red blood cells (RBC), hemoglobin (HGB), platelets (PLT), hematocrit (HCT), mean vascular volume (MCV) and mean vascular hemoglobin (MCH) (n = 5). (C) Hematoxylin & eosin (H&E) staining of major organs in mice sacrificed different days post injection of M@P-PDR. (Controls were mice injected with 0.9% aqueous sodium chloride solution.) Reference ranges of hematological data can be found from Wuhan Servicebio Laboratory (http://www.servicebio.cn).


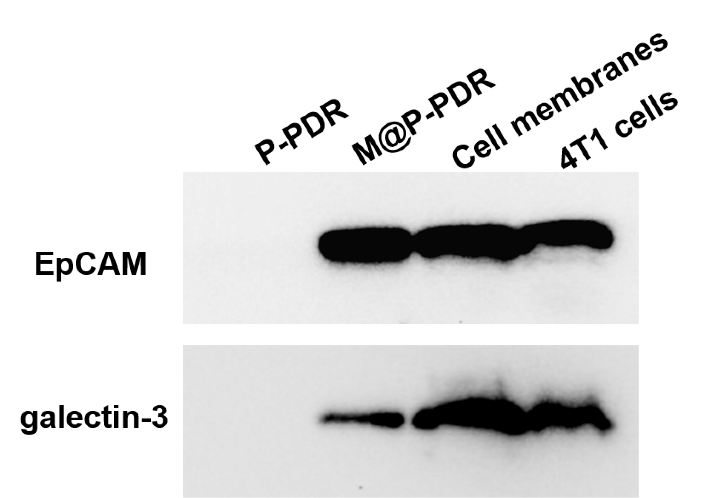


**Fig. S7.** Western blotting analysis of membrane-specific protein markers.


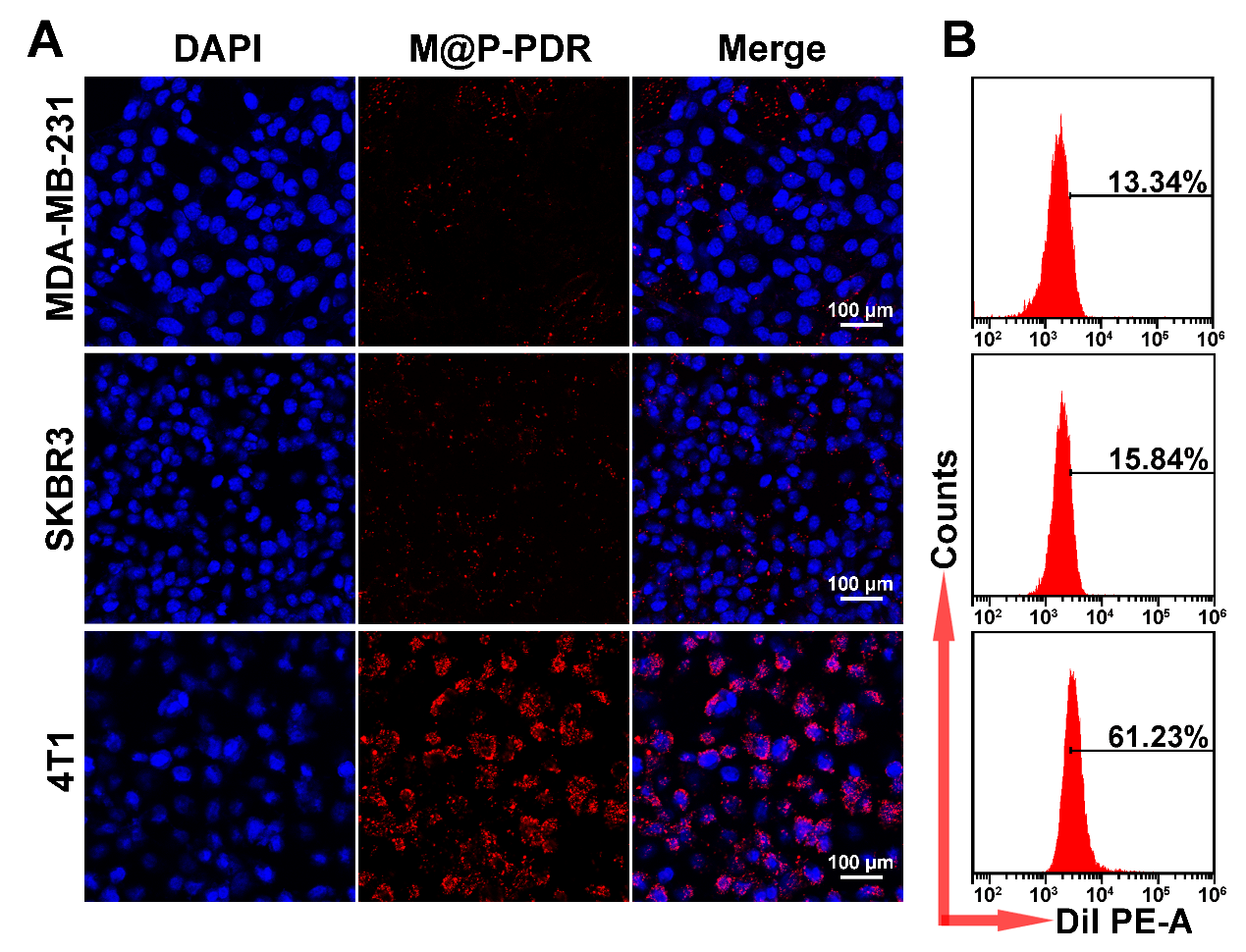


**Fig. S8.** Validation Homologous Targeting Capability of M@P-PDR. (A) CLSM images of MDA-MB-231 cells, SKBR3 cells or 4T1 cells, coincubated with M@P-PDR for 2h, respectively. (B) The corresponding flow cytometry quantitative analyses of intracellular uptake.


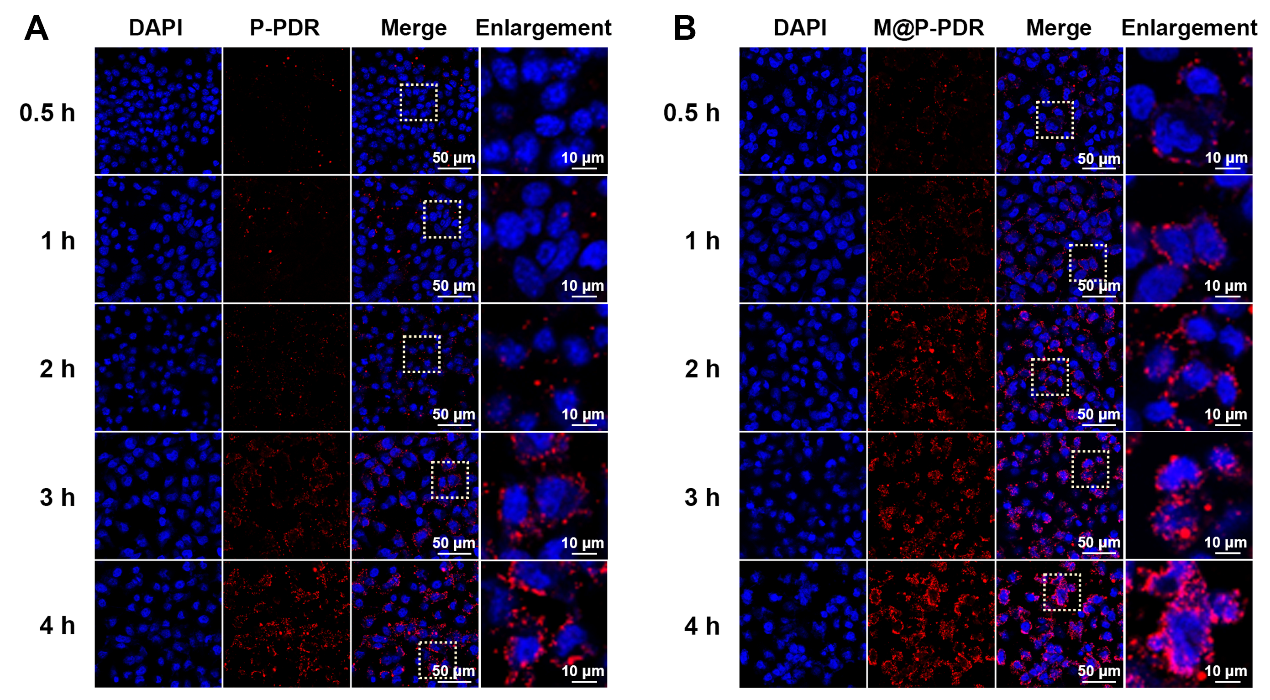


**Fig. S9.** (A) Confocal microscopy images of 4T1 cells treated with P-PDR and (B) M@P-PDR for different times (0.5, 1, 2, 3 and 4 h), respectively.


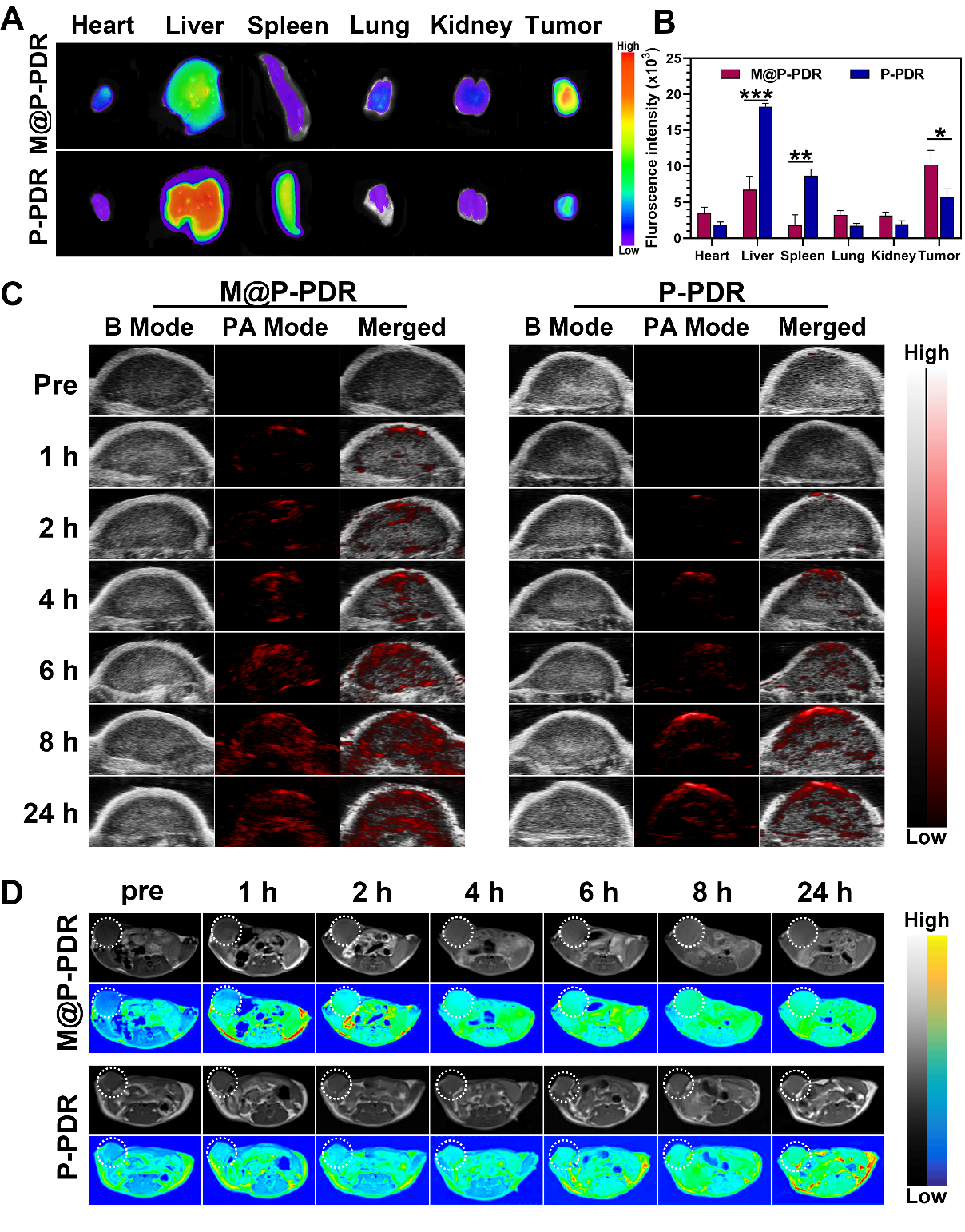


**Fig. S10.** (A) Fluorescence images of major organs and tumors in M@P-PDR and P-PDR treated groups 24 h after intravenous administration and (B) the corresponding fluorescence intensities (n = 3, t-test, *p < 0.05, **p < 0.01, ***p < 0.001, ****p < 0.0001). (C) *In vivo* PA images of tumor regions at different time points (pre-injection, 1, 2, 4, 6, 8 and 24 h) after injection of M@P-PDR and P-PDR. (D) T1-weighted MR images of 4T1 tumor-bearing mice at different time points.


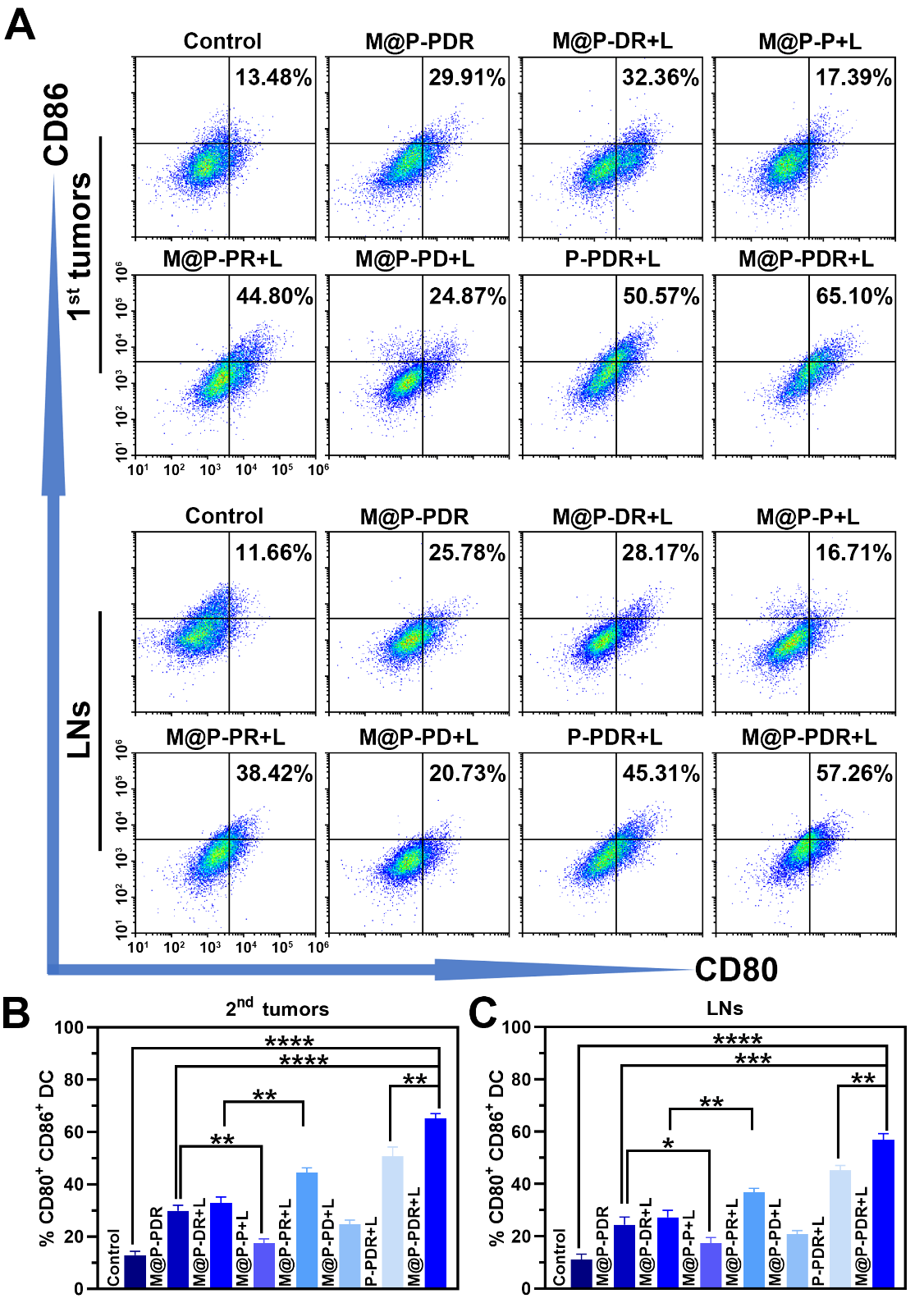


**Fig. S11.** (A) Flow cytometric analysis of DCs maturation in distant tumors (2^nd^) and lymph nodes in different groups. (B) The corresponding quantification of DCs maturation in distant tumors (2^nd^) and (C) the corresponding quantification analysis of DCs maturation in lymph nodes (LNs) in different groups (n = 3, t-test, *p < 0.05, **p < 0.01, ***p < 0.001, ****p < 0.0001).


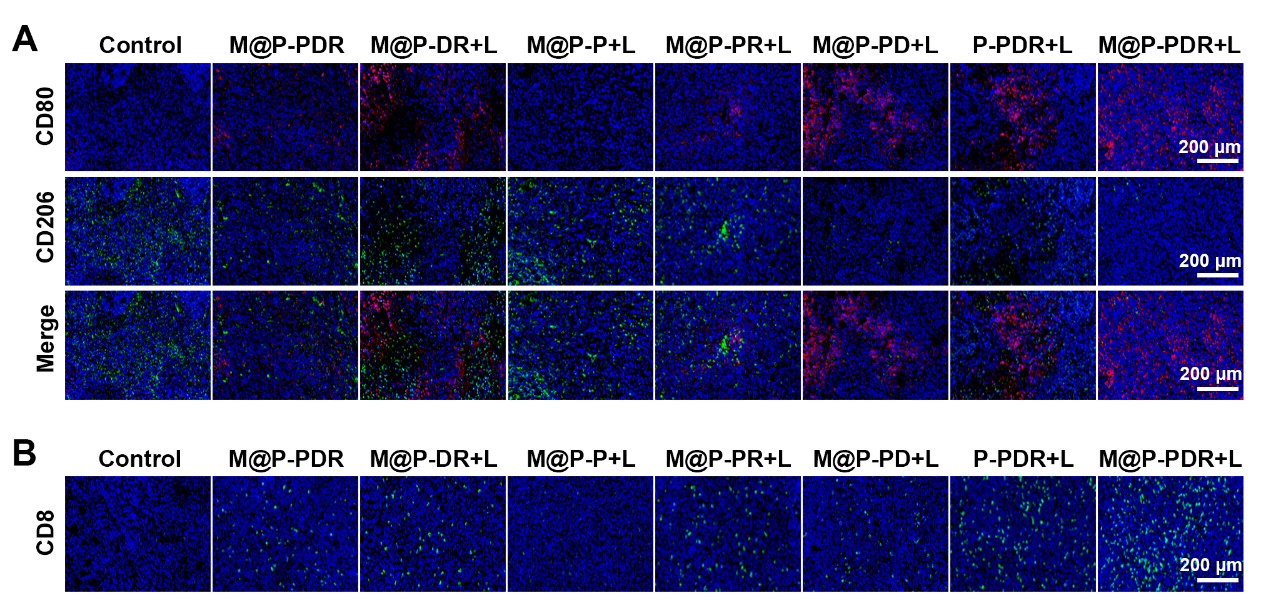


**Fig. S12.** (A) Immunofluorescence images of CD80 (red) and CD206 (green) in distant tumors (2^nd^) on day 9 after different treatments. (B) Immunofluorescence images of CD8^+^ T cells (green) in the distant tumors (2^nd^) on day 9 after different treatments. (C) H&E staining of the major organs (heart, liver, spleen, lung, kidney) of all groups collected on day 3 after different treatments.


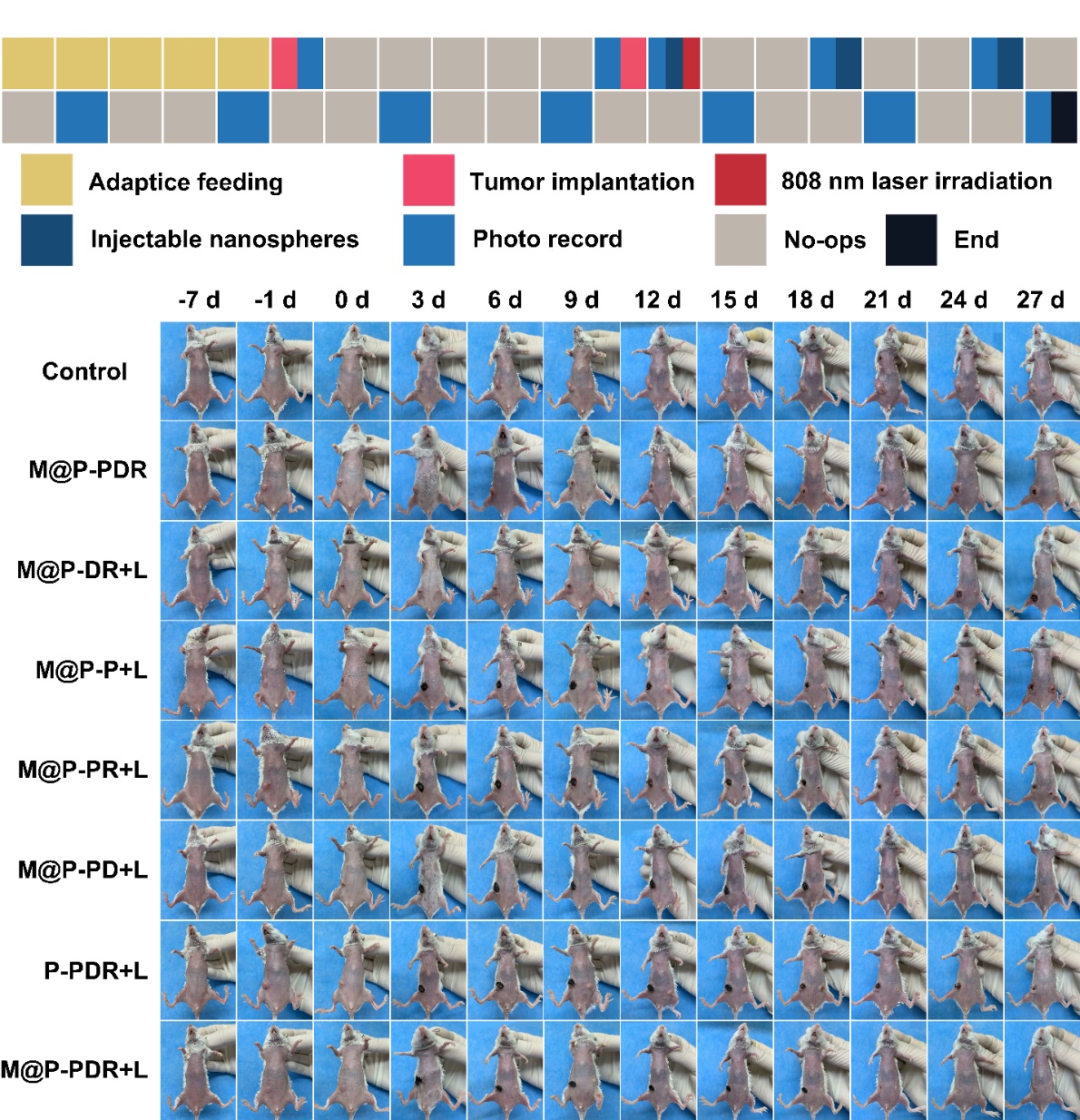


**Fig. S13.** (A) Photographic recording scheme for the 4T1 tumor-bearing mice (each square represents 1 day). (B) Images of mice during the 27-day treatment period.


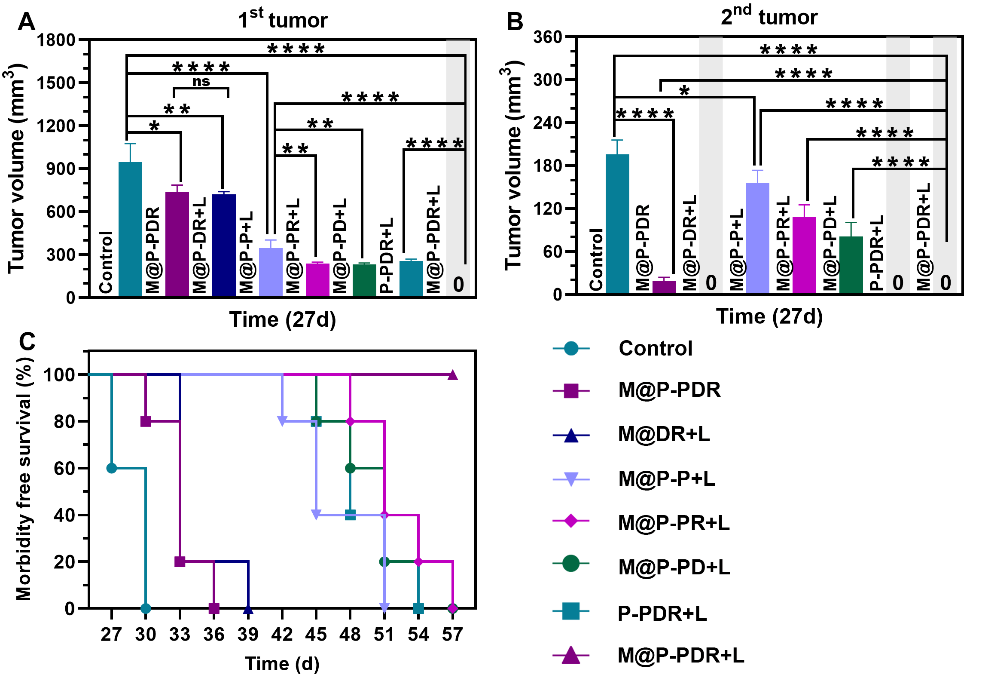


**Fig. S14.** (A) Statistical analysis of primary tumors (1^st^) and (B) distant tumors (2^nd^) on day 27 after different treatments (n = 5, t-test, *p < 0.05, **p < 0.01, ***p < 0.001, ****p < 0.0001). (C) Survival curves of mice after different treatments.


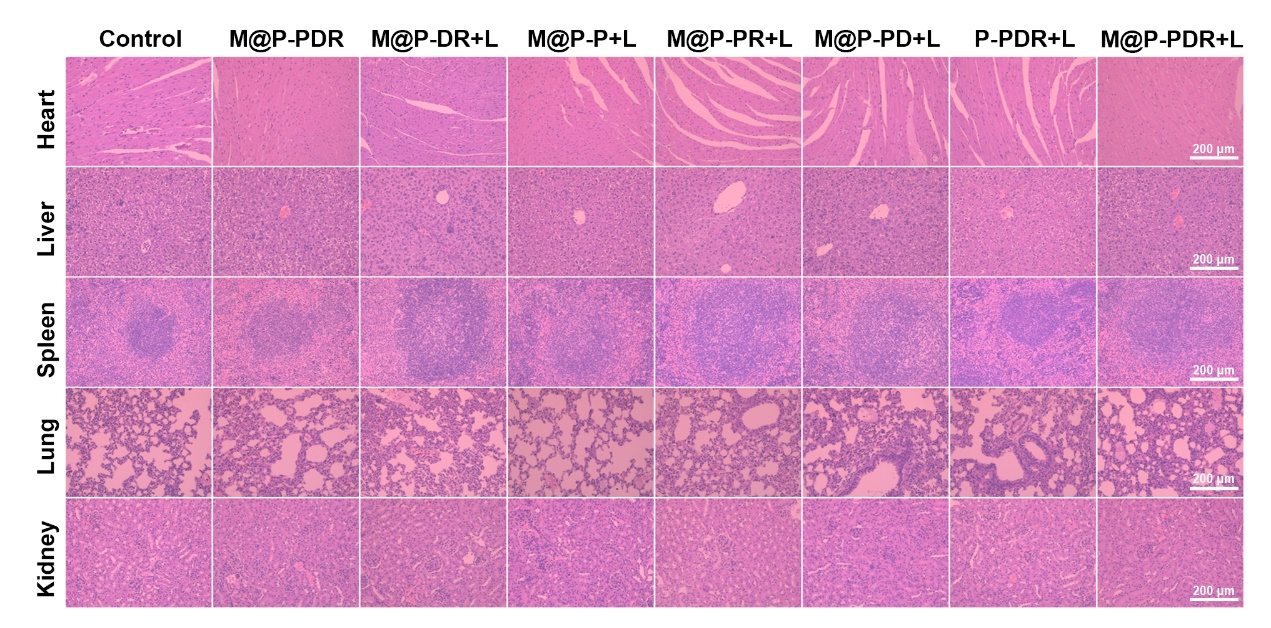


**Fig. S15**. H&E staining of the major organs (heart, liver, spleen, lung, kidney) of all groups collected on day 3 after different treatments.


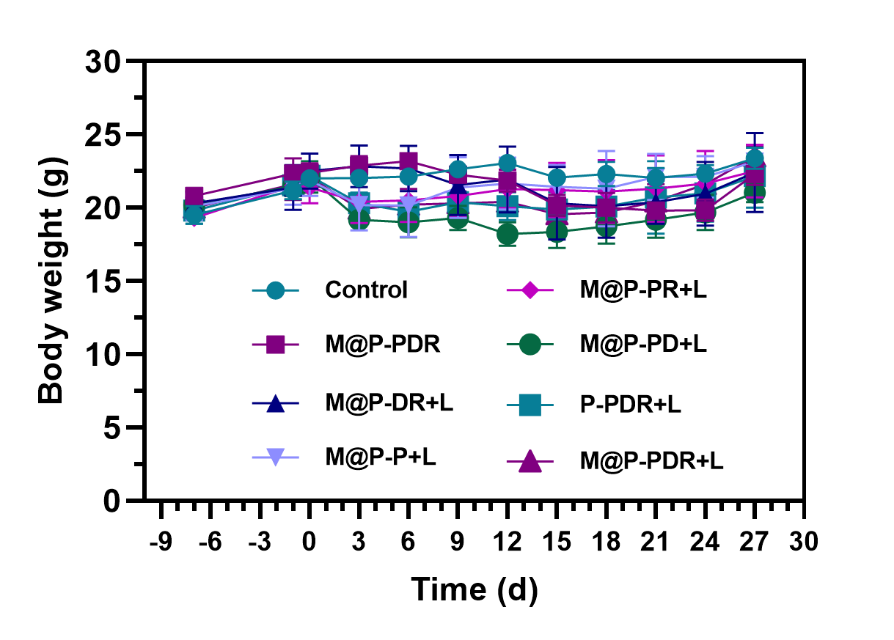


**Fig. S16**. Time-dependent body weight curves of mice in the different groups (n = 5).

**Tab.1 The Comparison of Photothermal/Chemo-/Immuno- Cocktail Therapy with other Tumor Therapeutic Modalities**

| **Treatment** | **Advantages** | **Disadvantages** | **Cases** | **Reference** |
| --- | --- | --- | --- | --- |
| Chemotherapy | Inhibit tumor cell proliferation. | Most chemotherapeutic drugs have dose-limiting toxicity and inefficient intracellular accumulation. | Paclitaxel (PTX) and Doxorubicin (DOX) | [3, 4] |
| Immunotherapy | Activate host defense to identify, attack, and eradicate cancer cells. | High economic cost, low immune response rate, large individual differences, circumscribed antitumor efficacy, and some immune-related adverse events (irAEs). | Immune checkpoint blockade (ICB) strategies, chimeric antigen receptor T cell engineering treatment, and cancer vaccines. | [5, 6] |
| PTT | Noninvasiveness, lower required power of laser irradiation and extremely low toxicity to normal tissues. | It cannot treat recurring and metastatic tumors. | PTAs (ICG, CuS)-based PTT | [7, 8] |
| PTT + Immunotherapy | Hyperthermic ablation of tumors can convert cancer cells from non-immunogenic to immunogenic. | Solid tumors usually possess immunosuppressive TME infiltrated with abundant immune suppressive cells, which causes ineffective antitumor immune responses and drug resistance to immunotherapy. | PTT + R837  PTT + cytosine-phosphate-guanine oligodeoxynucleotides (CpG ODN) | [9, 10] |
| Photothermal/Chemo-/Immuno- Cocktail Therapy | Cocktail therapy not only effectively ablates the primary tumor, but also reverses the immunosuppressive TME and enhances the antitumor efficacy. |  |  |  |

**References**

1. Liang X, Deng Z, Jing L, Li X, Dai Z, Li C, Huang M. Prussian blue nanoparticles operate as a contrast agent for enhanced photoacoustic imaging. Chem Commun. 2013;49:11029-31.
2. Busquets MA, Estelrich J. Prussian blue nanoparticles: synthesis, surface modification, and biomedical applications. Drug Discov Today. 2020;25:1431-43.
3. Das T, Anand U, Pandey SK, Ashby CR, Jr., Assaraf YG, Chen ZS, Dey A. Therapeutic strategies to overcome taxane resistance in cancer. Drug Resist Updat. 2021;55:100754.
4. Zeng Y, Zhang X, Lin D, Feng X, Liu Y, Fang Z, Zhang W, Chen Y, Zhao M, Wu J *et al*. A lysosome-targeted dextran-doxorubicin nanodrug overcomes doxorubicin-induced chemoresistance of myeloid leukemia. J Hematol Oncol. 2021;14:189.
5. Lee AH, Sun L, Mochizuki AY, Reynoso JG, Orpilla J, Chow F, Kienzler JC, Everson RG, Nathanson DA, Bensinger SJ et al. Neoadjuvant PD-1 blockade induces T cell and cDC1 activation but fails to overcome the immunosuppressive tumor associated macrophages in recurrent glioblastoma. Nat Commun. 2021;12:6938.
6. Usyk M, Pandey A, Hayes RB, Moran U, Pavlick A, Osman I, Weber JS, Ahn J. Bacteroides vulgatus and Bacteroides dorei predict immune-related adverse events in immune checkpoint blockade treatment of metastatic melanoma. Genome Med. 2021;13:160.
7. Ting CW, Chou YH, Huang SY, Chiang WH. Indocyanine green-carrying polymeric nanoparticles with acid-triggered detachable PEG coating and drug release for boosting cancer photothermal therapy. Colloids Surf B Biointerfaces. 2021;208:112048.
8. Zhang M, Qin X, Xu W, Wang Y, Song Y, Garg S, Luan Y. Engineering of a dual-modal phototherapeutic nanoplatform for single NIR laser-triggered tumor therapy. J Colloid Interface Sci. 2021;594:493-501.
9. Jiang F, Ding B, Liang S, Zhao Y, Cheng Z, Xing B, Ma P, Lin J. Intelligent MoS(2)-CuO heterostructures with multiplexed imaging and remarkably enhanced antitumor efficacy via synergetic photothermal therapy/ chemodynamic therapy/ immunotherapy. Biomaterials. 2021;268:120545.
10. Ming J, Zhang J, Shi Y, Yang W, Li J, Sun D, Xiang S, Chen X, Chen L, Zheng N. A trustworthy CpG nanoplatform for highly safe and efficient cancer photothermal combined immunotherapy. Nanoscale. 2020;12:3916-30.
